# Plant-mediated nitrous oxide emissions

**DOI:** 10.3389/fpls.2026.1884101

**Published:** 2026-08-18

**Authors:** Aamer Mumtaz, John G. Hampton, Timothy J. Clough, David W. M. Leung, Hossein Alizadeh

**Affiliations:** 1 Faculty of Agriculture and Life Sciences, Lincoln University, Lincoln, New Zealand; 2 School of Biological Sciences, University of Canterbury, Christchurch, New Zealand

**Keywords:** ammonia-oxidizing microorganisms, endophytes, environment, microbes, nitrate metabolism, plant physiology, phyllosphere

## Abstract

Plants are increasingly recognized as active contributors to nitrous oxide (N_2_O) fluxes. However, the physiological and biochemical processes underlying these plant-mediated N_2_O emissions remain poorly defined. This review synthesizes current understanding of direct N_2_O production pathways within plants, including chloroplast- and mitochondrial-associated processes involving nitrate and nitrite reductases, as well as cytochrome-mediated reactions under specific conditions. Plant-microbe interactions in the rhizosphere and phyllosphere further influence N_2_O dynamics. Symbiotic nitrogen-fixing bacteria and ammonia-oxidizing organisms modulate nitrogen availability and transformation, altering net fluxes. Evidence is also growing for conduit-mediated N_2_O transport from roots to aerial tissues, as well as for foliar N_2_O uptake, suggesting plants may function as both sources and sinks depending on context. Methodological limitations in separating plant- from microbe-derived N_2_O are critically evaluated, with a focus on challenges in isotopic tracing and sterile growth conditions. Key plant traits and environmental drivers influencing emissions are identified, including leaf area, root length, stomatal density, and hypoxic stress. This review provides an integrated physiological and ecological perspective on plant-associated N_2_O fluxes and proposes a framework linking plant traits, microbial partnerships, and environmental conditions to N_2_O outcomes. Clarifying these mechanisms is essential for incorporating plant processes into global N_2_O budgets and mitigation strategies.

## 1 Introduction

The nitrous oxide (N_2_O) molecule has a global warming potential value of 273 times that of carbon dioxide over a 100-year time horizon and is the dominant ozone-depleting substance (Ravishankara et al., 2009; IPCC, 2023). Atmospheric N_2_O concentration continues to increase due to anthropogenic activities (e.g., from 321.8 ppb in 2018 to 336.7 ppb in 2023), averaging 0.78 ppb per annum over the last decade (World Meteorological Organization, 2024). Unlike other greenhouse gases, atmospheric N_2_O is not regulated under the Montreal Protocol (UN Environment Programme, 2013). Soil microbial activities following N fertilizer application or the return of animal excreta are responsible for around two-thirds of anthropogenic N_2_O emissions (Schlesinger, 2010; Hu et al., 2015; Tian et al., 2020; Ahmed et al., 2022). This has governed the approaches used towards reducing N_2_O emissions, primarily through soil and pasture management and associated microbiological processes (Smith and Conen, 2004). However, lichens and plants can also be a source of N_2_O (Smart and Bloom, 2001; Yu and Chen, 2009; Lenhart et al., 2015; Timilsina et al., 2020b; Qin et al., 2024). Plants play an important role in the nitrogen cycle through N uptake, storage, and interactions with microbial nitrogen transformations (Fowler et al., 2013; Abalos et al., 2014b). Increasing evidence indicates that plants influence atmospheric N_2_O exchange through internal production, microbial associations, conduit-mediated transport, and foliar uptake (Qin et al., 2024). These emissions can be from leaves (Smart and Bloom, 2001) or stems (Díaz-Pinés et al., 2016) or from plants acting as a conduit for N_2_O produced by bacteria and fungi in the soil (Chang et al., 1998). Consequently, substantial uncertainty remains regarding both plant coverage and emission parameters. Potentially, this has implications for developing N_2_O mitigation methods (Bastviken et al., 2023), because the management of N in agricultural ecosystems will also need to consider including plants in addition to soil-derived N_2_O. Understanding the mechanisms underlying plant-associated N_2_O emissions is essential for accurately quantifying their contribution to ecosystem and global N_2_O budgets.

Previous reviews have examined individual aspects of plant-associated N_2_O emissions, including nitrate reductase activity, conduit transport, and microbial contributions (Sun et al., 2018; Lenhart et al., 2019; Timilsina et al., 2022a) However, a synthesis integrating plant physiology, microbial interactions, transport processes, and environmental controls is currently lacking. This review aims to provide that integrated perspective and identify key knowledge gaps and future research priorities.

In this review, the term plant-associated N_2_O emissions refers collectively to (i) internal plant-derived N_2_O production occurring within plant tissues, (ii) plant-mediated transport of soil-derived N_2_O through vascular or gas-conducting tissues, and (iii) N_2_O produced by microorganisms associated with plants, including rhizosphere, phyllosphere, and endophytic communities. Accordingly, a distinction is made between N_2_O production, where plants or microorganisms directly generate N_2_O, and N_2_O mediation, where plants influence the transport, release, or microbial production of N_2_O without necessarily producing it themselves. Where possible, these processes are distinguished throughout the review to avoid conflating plant metabolic activity with microbial N_2_O production.

## 2 N_2_O sources and the emerging role of plants

Globally, emissions of N_2_O to the atmosphere are currently 60% from natural sources and 40% from anthropogenic sources (Tian et al., 2020; World Meteorological Organization, 2022; World Meteorological Organization, 2024). Agricultural sources of N_2_O equate to ~ 73% of total anthropogenic N_2_O emissions and result from tillage practices, N fertilizer application, and livestock excreta (Venterea et al., 2011; Hockstad and Hanel, 2018). However, substantial uncertainties remain in quantifying natural N_2_O sources, particularly from unmanaged ecosystems. Plant-mediated N_2_O emissions, which are often overlooked or underestimated in global inventories, could represent a significant but poorly constrained component of these natural fluxes (Qin et al., 2024). An improved understanding of how plants contribute as direct emitters and conduits or sinks for N_2_O could help narrow these uncertainties and enhance the accuracy of global N_2_O budget models, particularly considering climate change and shifting land-use patterns.

### 2.1 Magnitude of N_2_O

The emission of N_2_O from soil is well recognized, primarily occurring because of microbial nitrification and denitrification processes (Schlesinger, 2010; Butterbach-Bahl et al., 2013; Hu et al., 2015; World Meteorological Organization, 2024). However, plants can also be a source of N_2_O emissions (**Table 1**). Smart and Bloom (2001) reported that wheat (*Triticum aestivum*) leaves can produce N_2_O at a rate of <316.8 µg N_2_O-N/m²/h due to photo-assimilation in the chloroplast. This is the smallest amount of trace gas discovered for higher species of plants. Qin, Pang (Qin et al., 2024) studied 52 different trees, shrubs, and herbs *in situ* and found that they were capable of producing N_2_O from their leaves. Gogoi and Baruah (2012), who investigated N_2_O emissions from wheat and rice fields using a closed chamber technique, found that N_2_O emissions varied among varieties. In wheat varieties, N_2_O emissions ranged from 12 to 291µg N_2_O-N/m²/h, while in rice (*Oryza sativa*) varieties they ranged from 11 to 154 µg N_2_O-N/m²/h. Additionally, the rate of emissions varied with soil temperature: increasing temperature enhanced enzymatic activities and soil respiration, ultimately increasing denitrification and N_2_O emissions (Smith, 1997). For wheat and rice there was also a positive correlation between N_2_O emissions and both root biomass and leaf area (Gogoi and Baruah, 2012). Dean and Harper (1986) studied N_2_O emissions in soybean (*Glycine max*) and winged bean (*Psophocarpus tetragonolobus*) plants (**Table 1**), providing strong evidence that nitrate reductase activity is closely associated with N_2_O formation in these species. In soybean leaves, emissions reached approximately 2520 ng N_2_O-N/g/h, while winged bean leaves emitted around 20,716 ng N_2_O-N/g/h. These emissions were derived from ¹^5^N-labelled nitrate (NO_3_^-^), and importantly, no N_2_O was detected in NR-deficient soybean mutants, providing strong evidence that NR activity is essential for N_2_O formation in these species. Lenhart, Behrendt (Lenhart et al., 2019) studied 32 plant species to determine if they produced N_2_O emissions using either NO_3_^-^, ammonia (NH_3_), or glycine as precursors. In sterile conditions N_2_O production ranged from 4.7 to 151 ng N_2_O/g/h, while in non-sterile conditions N_2_O emissions ranged from 0.4 to 173 ng/g/h. The authors suggested that in sterile conditions, plants may produce more N_2_O because no microbes are competing for N or consuming the N_2_O they release. In non-sterile environments, microbes might utilize N or break down the N_2_O, so that the plant’s emissions appear lower (Bano et al., 2024). Hakata, Takahashi (Hakata et al., 2003) reported a 58-fold difference in N_2_O emissions among 17 plant species, with emissions ranging from -0.012 ± 0.12 ng N_2_O/g/h in manna gum (*Eucalyptus viminalis)* to 0.45 ± 0.20 ng N_2_O/g/h in Kenaf (*Hibiscus cannabinus*). Earlier, a 600-fold variation was reported among 217 plant species (Morikawa et al., 1998). Li, Lee (Li et al., 2011) found significant N_2_O emissions from cotton (*Gossypium hirsutum*) and soybean plants under field conditions (**Table 1**). After fertilizer application, N_2_O emissions from cotton ranged from 27.4-34.4 µg N_2_O-N/m²/h, and in soybeans, from 10.2 - 26.1 µg N_2_O-N/m²/h. The fluxes of N_2_O emissions were greater for cotton in daylight than at night, from which it can be assumed that stomatal exchange influenced the N_2_O emissions, while for soybean, N_2_O emissions in daylight did not differ from those at night. The latter was also reported by Qin, Pang (Qin et al., 2024), who found no significant differences in N_2_O production between dark and light conditions for different trees, shrubs, and herbs. Chen, Boeckx (Chen et al., 1999) studied N_2_O emissions from perennial ryegrass (*Lolium perenne*) *in situ* and reported a range of 0 to 116.7 µg N_2_O-N/m²/h (**Table 1**). After comparing total emissions with soil emissions, they concluded that perennial ryegrass could emit N_2_O. N_2_O emissions have also been observed from plants even under sterile conditions (Dean and Harper, 1986; Smart and Bloom, 2001; Lenhart et al., 2019), confirming that plants can produce N_2_O independently of microbial activity. Nonetheless, studying plants in the absence of microbes remains essential for accurately isolating and understanding the specific biochemical pathways and mechanisms responsible for plant-derived N_2_O emissions. A short summary of the N_2_O emissions from the above-mentioned studies is presented in **Table 1**.

**Table 1:** N_2_O emissions from different crops.

Crop	N_2_O emissions	Reference
Soybean	2520 ng N_2_O-N/g/h	Dean and Harper (1986)
Winged bean	20,716 ng N_2_O-N/g/h	Dean and Harper (1986)
Soybean	14.1 ng N_2_O-N/g/h and 19.7 ng N_2_O-N/g/h	Lifeng et al. (2000)
Manna gum	-0.012 ± 0.12 ng N_2_O-N/g/h	Hakata et al. (2003)
Kenaf	0.45 ± 0.20 ng N_2_O-N/g/h	Hakata et al. (2003)
Beech Forest (*Fagus sylvatica)*	1.27 to 6.36 ng N_2_O-N/g//h	Pihlatie et al. (2005)
52 different trees, shrubs and herbs	3.2 to 9.2 ng N_2_O-N/g/h	Qin et al. (2024)
32 plant species	4.7 to 151 ng N_2_O-N/g/h and 0.4 to 173 ng N_2_O/g/h	Lenhart et al. (2019)
Perennial ryegrass	0 to 116.7 µg N_2_O-N/m²/h	Chen et al. (1999)
Wheat	<316.8 µg N_2_O-N/m²/h	Smart and Bloom (2001)
Soybean	10.2 to 26.1 µg N_2_O-N/m²/h	Li et al. (2011)
Cotton	27.4 to 34.4 µg N_2_O-N/m²/h	Li et al. (2011)
Wheat	12 to 291 µg N_2_O-N/m²/h	Gogoi and Baruah (2012)
Rice	11 to 154 µg N_2_O-N/m²/h	Gogoi and Baruah (2012)
Riparian forest	−40 to 1500 µg N_2_O-N/m²/h	Mander et al. (2021)
Canola (*Brassica napus)*	0.45 mL/L	Chang et al. (1998)

Where possible, N_2_O emission values were converted to common units to improve comparability. Values that could not be reliably converted are presented in the original units reported by the authors. Differences in units reflect variations in experimental design and measurement approaches; therefore, values should be interpreted as indicative ranges rather than directly comparable emission rates.

Taken together, reported plant N_2_O emission measurements span several orders of magnitude largely because studies differ in units of expression, plant parts assessed (e.g., leaves, roots, or whole plants), environmental conditions, and the extent to which soil and microbial contributions are included. Consequently, these values should be interpreted as indicative ranges rather than directly comparable emission factors. Across studies, plant N_2_O fluxes range from near-zero emissions or even net uptake to substantial releases under specific conditions. Rather than reflecting inconsistency, this variability primarily arises from differences in species traits, environmental context, and experimental design.

Controlled laboratory assays using isolated leaves or seedlings e.g., Dean and Harper (1986) and Smart and Bloom (2001), typically report low N_2_O fluxes that are more indicative of direct plant metabolic processes. In contrast, field-based measurements at the crop or ecosystem scale [e.g., Gogoi and Baruah (2012), Li et al. (2011)] often yield higher fluxes, reflecting the integrated effects of plant activity, soil nitrogen availability, and rhizosphere microbial processes.

Species-specific physiological traits further contribute to this divergence. Plants characterized by high nitrate uptake capacity or elevated nitrate reductase activity can exhibit greater N_2_O production, as demonstrated by exceptionally high foliar emissions in winged bean, whereas other species, such as Manna gum, have shown negligible or negative net fluxes, suggesting potential N_2_O uptake under certain conditions (Rusch and Rennenberg, 1998; Lenhart et al., 2015). Environmental drivers modulate these responses by altering both plant metabolism and microbial N transformations. For example, temperature and soil conditions can stimulate microbial N_2_O production in the rhizosphere (Gogoi and Baruah, 2012) which may subsequently be transported through plant tissues and released to the atmosphere. Diurnal patterns also vary among species: some exhibit higher daytime emissions coinciding with stomatal opening (Li et al., 2011) while others show little or no diurnal variation (Qin et al., 2024).

Methodological context is therefore critical when interpreting reported fluxes. Comparisons between experiments conducted in sterile systems and those using natural soils highlight the extent to which microbial processes may either augment plant-associated N_2_O production or consume N_2_O before emissions. Collectively, these findings indicate that context-dependent interactions among plant physiology, microbial activity, and environmental conditions best explain apparent contradictions in the literature. Recognizing the roles of plant physiology, microbial activity, and environmental conditions in regulating N_2_O flux is essential for improving comparability among studies, refining measurement approaches, and constraining estimates of plant-mediated contributions to the global N_2_O budget. Plants are active components of ecosystem N_2_O cycling. Although the relative contribution of plant-associated pathways remains uncertain, they can influence N_2_O exchange through direct production, microbial interactions, transport processes, and atmospheric uptake.

### 2.2 Species-specific approach toward N_2_O emissions

Variations in plant root biomass, plant growth rates, and nutrient absorption pathways can lead to differences in N_2_O emissions from soils and wetlands (de Klein et al., 2020; Wang et al., 2024). Barneze, Petersen (Barneze et al., 2024) found that while longer and denser root systems can increase N_2_O emissions by enhancing soil exploration, greater total root biomass was associated with reduced soil NO_3_^-^ concentrations and lower N_2_O emissions. They attributed this to improved N uptake by plants, which decreases NO_3_^-^ availability for microbial processes that produce N_2_O.

Additionally, some plants have been reported to inhibit nitrification or denitrification processes, which can lead to decreased N_2_O emissions (Tiemeyer et al., 2020). For example, in winter, lucerne (*Medicago sativa*) and plantain (*Plantago lanceolata*) reduce N_2_O emissions compared to more traditional temperate pasture species, like perennial ryegrass and white clover (*Trifolium repens*), when growing in ruminant urine patches, although the mechanisms underlying this observed reduction in emissions have not been demonstrated (Luo et al., 2018). Plantain roots contain anti-microbial secondary metabolites (phenylpropanoid glycoside verbascoside and aucubin) (Herbert et al., 1991; Bartholomaeus and Ahokas, 1995), and their exudates can alter soil moisture, pH and nutrient availability, which is believed to inhibit nitrification, causing a reduction in N_2_O emissions (Simon et al., 2019). Several plants have been reported to possess the ability to suppress nitrification in soil. Among them are Brazilian-licorice (*Periandra mediterranea*) (Nogueira et al., 2019), mammoth wild rye (*Leymus racemosus*), gamba grass (*Andropogon gayanus*), molasses grass (*Melinis minutiflora*), groundnut (*Arachis hypogaea*), Italian ryegrass (*Lolium perenne* subsp*. multiflorum*) (Subbarao et al., 2007), drumstick tree (*Moringa oleífera*) (Elrys et al., 2019), thatching grass (*Hyparrhenia diplandra*) (Lata et al., 2000), Caucasian fir (*Abies nordmanniana*), Norway spruce (*Picea abies*) (Harter et al., 2016) and Guinea grass (*Megathyrsus maximus*) (Arp et al., 2002). Suppression of nitrification was only observed in the above-mentioned species when the source of N was ammonium (NH_4_^+^), and no suppression was observed when the source was NO_3_^-^ (Subbarao et al., 2007). A plant species combination (*Festuca arundinacea* and *Phleum pratense* L.) was also shown to be able to reduce N_2_O emissions, working as a sink for N_2_O (Abalos et al., 2014a).

In the following sections, this review compiles and discusses current knowledge of plant N_2_O emissions, highlighting the roles of plant traits, microbial interactions, and environmental factors, to better understand the significance of plants in the global N_2_O budget.

## 3 Mechanisms of N_2_O emissions from plants

### 3.1 Biochemical production pathways

Although plants have long been viewed as passive participants in the N cycle, they can actively mediate N_2_O emissions through multiple physiological pathways. These mechanisms extend beyond mere soil-microbe interactions, and encompass direct biochemical production within plant tissues, transport of soil-derived N_2_O via vascular systems, and even foliar absorption of atmospheric N_2_O (Lensi and Chalamet, 1981; Lenhart et al., 2019; Qin et al., 2024). The diversity of these pathways, often species- and environment-specific, complicates our understanding of plant contributions to N_2_O fluxes. This section explores the current mechanistic insights into plant-mediated N_2_O emissions, highlighting the key processes and knowledge gaps that must be addressed to refine emission models and mitigation approaches.

#### 3.1.1 Chloroplast and mitochondrial mechanisms

Plant-mediated N_2_O production can occur internally through biochemical pathways in specialized organelles, primarily chloroplasts and mitochondria.

**Figure 1:**
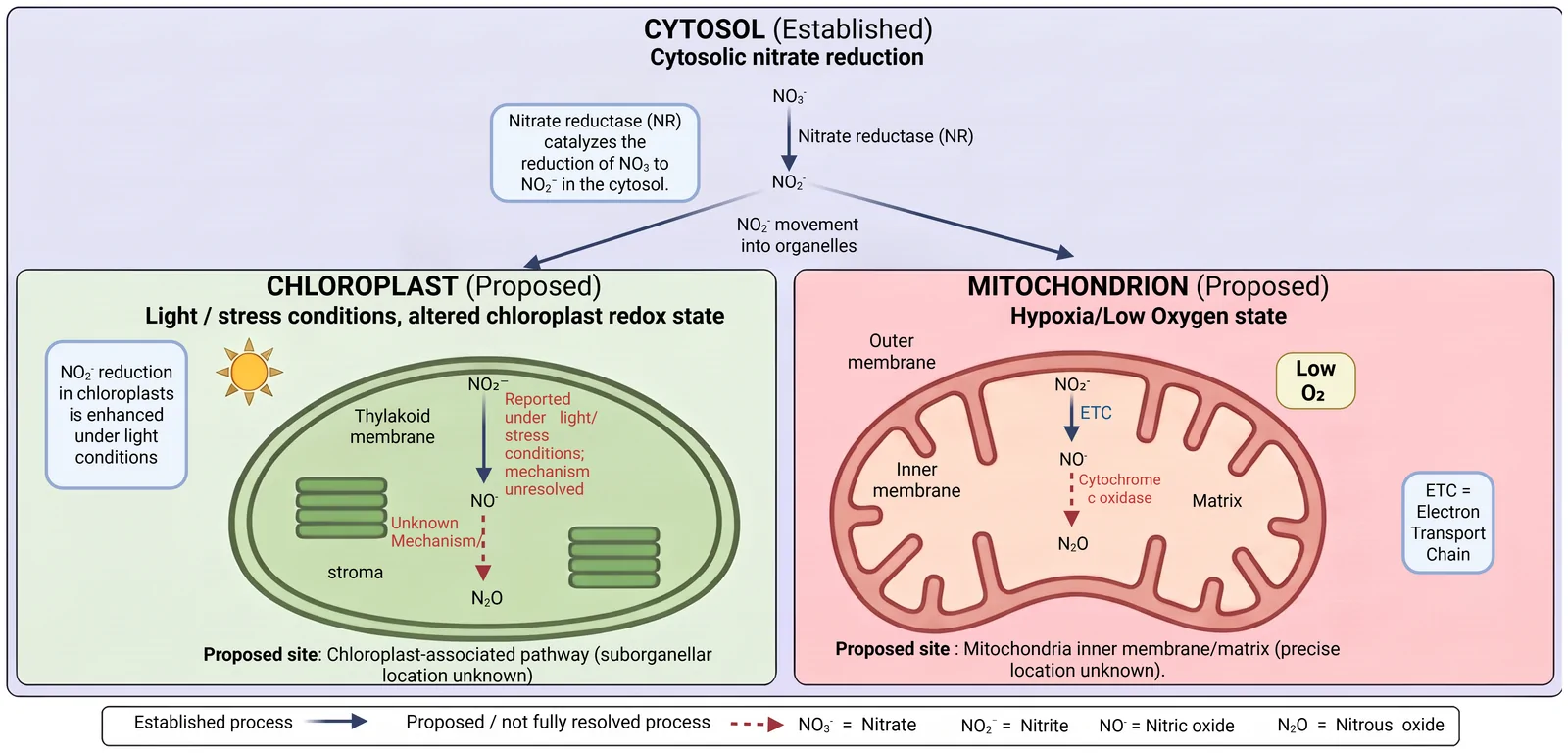
Conceptual schematic diagram of proposed pathways for N_2_O production in plants. Cytosolic nitrate reduction is well established (Dean and Harper, 1986; Smart and Bloom, 2001). Chloroplast-associated pathways are based on reports linking nitrate assimilation, nitrite metabolism and NO formation under light and stress conditions (Miflin, 1974; Smart and Bloom, 2001; Timilsina et al., 2022a). Mitochondrial pathways are based on evidence for nitrite reduction and NO metabolism under hypoxia (Brandão and Sodek, 2009; Oliveira and Sodek, 2013; Gupta et al., 2016), together with recent evidence for plant NO production under low-oxygen conditions (Timilsina et al., 2020b). Dashed red arrows indicate proposed or unresolved processes. Created in https://BioRender.com.

Light affects plant metabolism, influencing processes like photosynthesis and NO_3_^-^ reduction. In wheat, N_2_O production has been linked to light-dependent processes like photo-assimilation (Smart and Bloom, 2001; Bo et al., 2023). Nitrate reductase (NR), located in the cytoplasm, converts NO_3_^-^ to nitrite (NO_2_^-^), which is further processed by nitrite reductase (NiR) within the chloroplasts (Miflin, 1974). Smart and Bloom (2001) extracted these enzymes and intact chloroplasts. They found that NO_2_^-^ accumulated within chloroplasts and suggested that NO_2_^-^ might then be transformed into N_2_O. They showed light-dependent N_2_O formation associated with chloroplast NO_3_^-^/NO_2_^-^ reduction, but the biochemical step converting NO_2_^-^ to N_2_O remains unresolved; this pathway should therefore be regarded as a plausible but still mechanistically uncharacterized hypothesis rather than an established route (**Figure 1**). Lenhart, Behrendt (Lenhart et al., 2019) showed that N_2_O can still be emitted in the dark, suggesting alternative, non-light-dependent pathways for N_2_O production.

The apparent contradiction between light-dependent and dark N_2_O production likely reflects the operation of multiple pathways rather than mutually exclusive mechanisms. Smart and Bloom (2001) examined chloroplast-associated N_2_O formation linked to photosynthetic nitrate assimilation, whereas Lenhart, Behrendt (Lenhart et al., 2019) evaluated a wider range of species under different environmental conditions and demonstrated that N_2_O production can occur in darkness. Species-specific physiology, nitrogen availability, nitrite accumulation, oxygen status, and microbial contributions may all influence the relative importance of these pathways, with these factors discussed in greater detail in subsequent sections of this review. Further studies combining isotope tracing, sterile culture systems, and organelle-specific approaches are required to quantify the contribution of each mechanism. Timilsina, Oenema (Timilsina et al., 2022b) studied the relationship between rubber plant respiration and N_2_O emissions using ^15^N and found that plants were both a source and sink for N_2_O, with mitochondria proposed as the production site. Mitochondria contain the cytochrome c oxidase enzyme in their inner membrane. This enzyme is reported to have evolved from a denitrifying bacterium (Saraste and Castresana, 1994). The primary function of this enzyme is the reduction of oxygen (O_2_) to water (H_2_O) (Collman et al., 2007; Blomberg and Ädelroth, 2018). It has been proposed that cytochrome c oxidase and associated mitochondrial processes may contribute to the conversion of NO to N_2_O under hypoxic conditions (Collman et al., 2007; Blomberg and Ädelroth, 2018). There is also evidence to show that mitochondria in plant roots convert NO into N_2_O under anoxic and hypoxic soil conditions. This might be a defense mechanism as NO affects the signaling and respiratory mechanisms of the mitochondria (Poderoso et al., 2019). Under stress-free conditions, NO_3_^-^ in the cytosol is metabolized to NH_4_^+^ and then to amino acids (Brandão and Sodek, 2009), but under anoxic and hypoxic conditions, NH_4_^+^ production is suppressed and NO_3_^-^ is metabolized to NO_2_^-^ in the cytosol and then the accumulated NO_2_^-^ in the cytoplasm is transported to the mitochondria, where it is reduced to NO (Gupta et al., 2016). As discussed above the NO molecule can be lethal for mitochondria, and it has been proposed that mitochondria convert NO to N_2_O (Timilsina et al., 2022b). Although cytochrome c oxidase and mitochondrial NO detoxification can convert NO to N_2_O under hypoxic conditions, current evidence is mostly qualitative and does not yet quantify how large this flux is relative to total plant N loss. Collectively, these findings suggest that at least two partially independent pathways operate: a light-coupled chloroplast route expressed under high photosynthetic activity, and a light-independent pathway linked to mitochondrial respiration and NO detoxification that can function in darkness and hypoxia (**Figure 1**; note that hypothesized steps are indicated with dashed arrows to denote their tentative nature).

#### 3.1.2 Leaf-level nitrate reductase pathway

A further biochemical route involves NR activity directly within leaves, contributing to N_2_O formation during NO_3_^-^ reduction processes. Dean and Harper (1986) reported N_2_O emissions from soybean and winged bean plants and proposed that N_2_O formation was associated with nitrate reductase (NR) activity through NO_2_^-^ and NO intermediates. Similar observations were later reported by Lifeng, Boeckx (Lifeng et al., 2000). However, while these studies support a link between NR activity and N_2_O formation, the precise biochemical mechanism by which NO is converted to N_2_O within plant tissues remains unresolved (Timilsina et al., 2020b). Plants lack the canonical microbial denitrification enzymes responsible for NO reduction (Bellido-Pedraza et al., 2022), suggesting that N_2_O formation may involve alternative enzymatic or non-enzymatic reactions. The herbicides 2,4-D and bromoxynil can cause NO_2_^-^ to accumulate in the leaves (Klepper, 1979). When these two herbicides were sprayed on three-week-old soybean leaves, N_2_O fluxes from the sprayed leaves were higher than from the unsprayed leaves. Nitrate accumulation was similar in sprayed and non-sprayed leaves, but NO_2_^-^ levels and N_2_O emissions were higher in sprayed leaves. Poderoso, Helfenberger (Poderoso et al., 2019) suggested that after herbicide application, the plant leaves converted NO_3_^-^ to NO_2_^-^, and since NO_2_^-^ and nitric oxide (NO) are toxic to plants, they proposed that soybean plants convert NO_2_^-^ and NO to N_2_O as a defense mechanism. However, the precise enzymatic or non-enzymatic mechanisms involved in NO-to-N_2_O conversion within plant tissues remain unclear. Recent studies, however, have challenged the assumption that NO_3_^-^ reduction in plants is solely driven by plant enzymes. Qin, Pang (Qin et al., 2024) suggested that NO_3_^-^ reduction in plants is not directly linked to photosynthetic electron transport. They proposed that it is likely driven by endophytes within plants that utilize photosynthates, such as glucose, as electron donors for NO_3_^-^ reduction (Timilsina et al., 2020b).

## 4 Plant-microbe interactions in the phyllosphere

The phyllosphere, especially leaves, hosts diverse microbial communities despite exposure to harsh conditions like UV radiation and desiccation (Vandenkoornhuyse et al., 2015). Adaptations such as pigment production and extracellular polysaccharides (EPS) help microbes survive and function on these surfaces (Sundin, 2002; Gal et al., 2003). The total leaf surface area of plants on Earth is estimated to be 64 million km^2^ in comparison to the Earth’s total surface area 510 million km^2^ (Morris and Kinkel, 2002). Ammonia oxidizing bacteria (AOB) and archaea (AOA) have been detected on plant leaf surfaces (the phyllosphere), and their role in N_2_O emissions is an emerging area of research.

Papen, Gessler (Papen et al., 2002) provided the first evidence that chemolithoautotrophic AOB and nitrite-oxidizing bacteria (NOB) colonize the phyllosphere of Norway spruce (*Picea abies*) in forests subjected to prolonged high atmospheric N deposition due to environmental pollution. These nitrifiers were predominantly located within the stomatal cavities of the needles. Field experiments demonstrated that the uptake of atmospheric NH_3_ by spruce needles was not solely a plant-driven process but also involved microbial activity. Specifically, the application of acetylene, an inhibitor of the ammonia monooxygenase enzyme in AOB, significantly reduced NH_3_ uptake, indicating that these bacteria actively contribute to NH_3_ assimilation within the needles. This study highlighted a previously unrecognized role of phyllosphere-associated nitrifiers in the N metabolism of forest trees under conditions of elevated N input. Similarly, Bowatte, Newton (Bowatte et al., 2015) found that AOB, predominantly *Nitrosospira* species, inhabit pasture grass leaves and oxidize NH_3_, producing N_2_O in the process. In their pasture experiments, leaf-associated AOB converted approximately 0.02–0.42% of NH_3_ into N_2_O (mean ~0.12%), indicating a small but significant flux. These leaf-surface AOB thus perform nitrification and, through processes like nitrifier denitrification or hydroxylamine oxidation, can release N_2_O, demonstrating that phyllosphere nitrifiers have the capacity to contribute directly to plant-associated N_2_O fluxes (Timilsina et al., 2022a).

Ammonia oxidizers inhabiting the phyllosphere rely primarily on externally supplied NH_3_ rather than plant-internal nitrogen sources (Papen et al., 2002; Bowatte et al., 2015). Atmospheric deposition, volatilized NH_3_ from fertilized soils or animal excreta, and nitrogen-rich aerosols provide substrates that can accumulate on leaf surfaces and within stomatal cavities (Papen et al., 2002; Fowler et al., 2013). Experimental studies indicate that only a small fraction of deposited NH_3_ is converted to N_2_O on leaf surfaces, typically less than 0.5% of total NH_3_ uptake (Bowatte et al., 2015). Consequently, phyllosphere nitrification is likely to represent a secondary N_2_O source relative to internal plant-associated microbial activity and soil-derived N_2_O transported through plants, with its importance greatest in nitrogen-enriched environments (Bowatte et al., 2015; Qin et al., 2024).

During preliminary research on the nitrification ability of detached leaves of perennial ryegrass from urine patches in the presence of NH_3_, a significant amount of NO_3_^-^ was recorded in the presence of NH_3_ (Bowatte et al., 2012). But it was not clear whether NO_3_^-^ was solely due to the presence of AOB on the leaves or because leaves were producing NO_3_^-^. The use of a nitrification inhibitor may be able to clarify this situation.

AOA have also been detected on foliage. Watanabe, Kohzu (Watanabe et al., 2016) confirmed the presence of AOA on Japanese cedar (*Cryptomeria japonica*) leaves and suggested they contribute to foliar nitrification. In that study, rainwater passing through the plant canopy collected AOA from leaf surfaces, resulting in detectable nitrification in the throughfall. In contrast, rainwater that did not contact foliage showed no nitrification activity, clearly demonstrating the role of canopy-dwelling AOA in N cycling.

In addition to surface colonizers, internal leaf endophytes have been identified as key agents of *in situ* N_2_O production. Qin, Pang (Qin et al., 2024) demonstrated that foliar N_2_O emissions can be strongly influenced by internal microbial communities, indicating that a substantial proportion of leaf-emitted N_2_O may originate from microbial processes within plant tissues rather than plant metabolism alone.

Given the widespread occurrence of this process, its potential contribution at the global scale has been increasingly recognized. Estimates suggest that foliar N_2_O emissions from endophytes may contribute approximately 1.0–1.2 Tg N_2_O-N per year, representing around 6–7% of total annual N_2_O emissions worldwide (Tian et al., 2024). However, these estimates remain subject to considerable uncertainty due to methodological limitations and challenges in distinguishing plant-derived from microbially derived N_2_O under natural conditions. Overall, these findings indicate that both leaf-surface microbes (AOB and AOA) and internal endophytes can actively contribute to N_2_O production, highlighting plants as important, but still not fully quantified, components of the global N_2_O budget.

Although AOB and AOA on leaf surfaces can produce N_2_O from deposited ammonia, available evidence suggests that this pathway contributes only a minor fraction of total plant-associated N_2_O emissions. Its relevance appears greatest in nitrogen-enriched environments and remains poorly quantified at ecosystem and global scales. Further work is needed to assess how widespread and environmentally significant phyllosphere nitrification is relative to other plant-associated N_2_O pathways. Quantifying the relative contributions of leaf-surface nitrifiers, endophytes, and plant-intrinsic processes remains a key challenge for accurately partitioning plant-associated N_2_O emissions.

## 5 Plant–rhizosphere interactions and their influence on N_2_O emissions

The term “rhizosphere” was originally defined by Lorenz Hiltner as the soil zone influenced by plant roots. Since then, it has attracted significant scientific interest across various disciplines as a key hotspot for interactions among microbes and between plants and microbes (Bakker et al., 2013). The interaction between plants, their rhizosphere microbial communities, and soil properties affects N_2_O emissions. The rhizosphere is a major site for denitrification, the microbial process that produces N_2_O as an intermediate or end product (Graf et al., 2022). Plant-derived organic carbon and fluctuating O_2_ availability levels in the plants’ roots stimulate the denitrification community (Henry et al., 2008). At the same time, roots influence nitrification by altering the distribution of NH_4_^+^ and O_2_ in soil. Consequently, soils with actively growing roots often develop hotspots of N transformations in the rhizosphere, which can either amplify N_2_O production or promote its reduction, depending on how plant-driven factors modulate microbial activity. The presence or absence of plants can lead to markedly different N_2_O fluxes from the soil (Ai et al., 2020), underlining that plant–microbe interactions are a central regulator of this greenhouse gas.

### 5.1 Root exudates and nitrifier communities

One key mechanism by which plants affect N_2_O emissions is through root exudates and nutrient uptake patterns that shape ammonia-oxidizing communities (both bacteria and archaea) responsible for nitrification (Bardgett et al., 2014; Finzi et al., 2015). AOB and AOA initiate the conversion of NH_4_^+^ to NO_3_^-^, releasing N_2_O in the process (either directly or indirectly via coupled nitrifier-denitrification). Plant-induced changes in soil pH and NH_4_^+^ availability can shift the balance between AOA and AOB (Coskun et al., 2017). For example, many plants prefer to absorb NO_3_^-^ and can make the soil around their roots more acidic or alkaline, depending on the form of N they use. This change in soil conditions can encourage certain nitrifying microbes to grow more than others (Saud et al., 2022). Perhaps the most prominent plant influence on nitrifiers is the production of biological nitrification inhibitors (BNIs) by certain species (Zakir et al., 2008; Nardi et al., 2013). Some crops exude compounds that specifically suppress AOB nitrification activity. Some of the examples are also discussed in subsection 1.2. Tropical grasses like *Brachiaria* have been shown to release potent BNIs that reduce nitrification rates (Subbarao et al., 2009). Likewise, the pasture herb plantain (*Plantago lanceolata*) exudes BNIs; incorporating plantain into grasslands has been demonstrated to curb soil N_2_O emissions in grazed systems by inhibiting nitrification in urine patches (Ding et al., 2025). Such plant-derived nitrification inhibition can lower the production of NO_3_^-^ and directly limit N_2_O formation by AOB. In the absence of these inhibitory exudates, plants that release abundant labile carbon (e.g. sugars, organic acids) may indirectly stimulate nitrifier populations. Ai, Zhang (Ai et al., 2020) found that wheat rhizodeposition induced a 3.5–9.2-fold increase in N_2_O emissions under certain conditions, suggesting that root exudates can increase nitrification or nitrifier-denitrification by providing energy sources to NH_3_ oxidizers. They attributed this rise to the indirect stimulation of microbial communities: root exudates fueled heterotrophic respiration, lowering O_2_ levels, increasing mineral N through microbial turnover, and creating microenvironments favorable to nitrifiers and denitrifiers. In this context, exudate-derived carbon serves as an “energy source” not by being consumed directly by AOB or AOA, but by shaping the rhizosphere in ways that promote their activity (Florio et al., 2017).

While AOB and AOA are autotrophic and rely on CO_2_ as their carbon source (Wang et al., 2023), studies show that labile carbon from root exudates indirectly supports their populations. For example, glucose amendments, which resemble simple root exudates, have been shown to alter nitrification dynamics and improve conditions for nitrifier activity. When glucose was added to paddy soil, a significant increase in nitrification-driven N_2_O production occurred under specific moisture conditions (Yang et al., 2019). This was not because the nitrifiers consumed the glucose, but because of heterotrophic microbial mineralization of the labile carbon. This process increases ammonium availability and changes redox conditions in the rhizosphere, creating favorable microenvironments for nitrifier growth and activity (Yao et al., 2022; Zhang et al., 2023). This indirect stimulation boosts nitrifier populations (AOA and AOB), increasing nitrification rates and potentially leading to higher N_2_O emissions.

While biological nitrification inhibition (BNI) traits clearly reduce soil-derived N_2_O emissions by suppressing nitrifier activity, their influence on plant-mediated N_2_O emissions remains less certain (Subbarao et al., 2007; Subbarao et al., 2009). BNI-producing species primarily lower N_2_O fluxes indirectly by limiting microbial NO_3_^-^ production in the rhizosphere, thereby reducing the pool of N_2_O available for root uptake or plant-mediated transport (Coskun et al., 2017). While many plant traits and microbial processes interact to regulate N_2_O dynamics, their combined effects remain insufficiently quantified across ecosystems. However, current evidence does not demonstrate that BNI traits directly suppress internal N_2_O production within plant tissues (Rusch and Rennenberg, 1998; Lenhart et al., 2015). As such, reductions in ecosystem-scale N_2_O emissions associated with BNI-active plants should not be interpreted as evidence of reduced plant metabolic N_2_O formation, but rather as a consequence of altered soil nitrogen transformations. In summary, BNI-producing plants effectively reduce soil microbial N_2_O production, but whether they also reduce plant-internal N_2_O emissions remains unknown.

### 5.2 Plant species, denitrifiers, and N_2_O production

Beyond nitrifiers, plant inputs of carbon and N substantially regulate denitrifying microbes in the rhizosphere. The denitrification community converts NO_3_- to N_2_O and N_2_, and they are typically heterotrophs stimulated by organic carbon (Graf et al., 2014). Root exudates (and decaying root tissues) serve as rich electron donors for the denitrification community, often accelerating N_2_O production under the O_2_ limited microsites around roots (Henry et al., 2008; Philippot et al., 2009). Different plant species release varying amounts and types of carbon into the soil, which influences the denitrifying microbial community (Baggs et al., 2023; Favela et al., 2023). For example, cereals and grasses tend to allocate more carbon to root exudation during growth, which can promote a higher proportion of denitrifiers in their rhizospheres (Hamonts et al., 2013; Ai et al., 2020). Legumes, on the other hand, influence soil N availability through biological N fixation and subsequent nodule and residue turnover. When legumes like soybeans are introduced into cropping systems, they alter the rhizosphere N cycle. Soybean roots release pulses of organic N from nodules and residues, which soil microbes convert into NH_4_^+^ that can then be nitrified (Saeki et al., 2017). Soybean is an example of a legume where nodule decomposition is a major source of N_2_O in the rhizosphere. As nodules age and break down, they release NH_4_^+^, which is first converted to NO_3_^-^ by nitrifiers and then partially converted to N_2_O by denitrifiers (Sánchez and Minamisawa, 2019). Interestingly, the soybean’s symbiotic bacteria (*Bradyrhizobium*) can also modulate the fate of this N_2_O. Many *Bradyrhizobium* strains possess a N_2_O reductase enzyme (nosZ) that can further reduce N_2_O to N_2_ (Sameshima-Saito et al., 2004; Sameshima-Saito et al., 2006). If nosZ-bearing strains dominate, they act as a N_2_O sink in the rhizosphere, but if strains lacking nosZ prevail, more of the N_2_O escapes to the atmosphere. This plant microbe interplay was demonstrated by Itakura, Uchida (Itakura et al., 2013), who reported that indigenous soybean bradyrhizobia without nosZ lead to net N_2_O emissions, whereas inoculating soybeans with engineered or selected mutated nosZ strains could significantly enhance N_2_O reduction to N_2_. Thus, the effect of a legume on N_2_O emissions depends not only on providing substrates for denitrification but also on the traits of its microbial partners. More generally, plant species can differ widely in how their rhizospheres either promote N_2_O production or absorption. In one comparative study by Shen, Huang (Shen et al., 2024), intercropping maize with soybean led to 16–41% lower N_2_O emissions than from maize alone. The reduction was attributed to more efficient overall N uptake and a suppression of AOB-driven nitrification in the intercropped soil. This example highlights that mixing plant species (or cropping sequences) can modulate microbial competition and substrate use in the rhizosphere, ultimately affecting N_2_O outcomes. Recent studies by Graf, Jones (Graf et al., 2022) with different crop species [e.g. barley (*Hordeum vulgare*) vs. sunflower] suggest that plant roots can help increase beneficial microbes around them, specifically, those that convert N_2_O into N, compared to microbes found in the rest of the soil. In other words, some plants create rhizosphere conditions that favor microbes capable of completing denitrification to N_2_, potentially mitigating N_2_O release. Overall, the plant species composition in an ecosystem, whether a N_2_-fixing legume, a high-carbon grass, or a particular crop genotype, exerts a strong influence on the denitrifier community and the balance between N_2_O production and reduction in soil.

### 5.3 Plant traits and symbioses modulating N_2_O

In addition to species identity, specific plant traits (physiological and morphological) play a crucial role in mediating N_2_O emissions. One important trait is plant N demand and uptake efficiency. Crops that rapidly take up available N (high N demand) leave less mineral N for microbial processes, thereby potentially lowering the substrate for nitrification and denitrification (Pan et al., 2022). When fertilizer N supply is well matched to crop demand, N_2_O emissions tend to be lower; conversely, when N inputs exceed plant needs, soil microbes convert the surplus into N_2_O at disproportionately high rates (Kelley et al., 2024). Field studies on wheat, for instance, show exponentially increasing N_2_O emissions once fertilizer levels surpass what the crop can utilize (Millar et al., 2018). Thus, high N use efficiency in plants can be viewed as a N_2_O-mitigating trait. Root architecture and depth are additional plant traits that significantly influence N_2_O emissions. Deep or extensive rooting can capture NO_3_- from deeper soil layers that might otherwise be denitrified to N_2_O (Aguilar et al., 2022). In wetland crops like rice, specialized root structures (aerenchyma) facilitate gas exchange between the plant and soil. Rice roots leak O_2_ into the surrounding soil and can also transport gases out (Steffens et al., 2011). This has a complex effect: the O_2_ stimulates nitrifiers in the immediate rhizosphere even under flooded conditions, leading to production of NO_3_^-^ (and thus potentially more N_2_O if that NO_3_^-^ is denitrified), but the same roots also create microsites of aerobic activity and even pathways for N_2_O to be reduced or vented as N_2_ (Arth et al., 1998). Experiments with aerenchyma-rich rice plants have shown that they can act as conduits, promoting coupled nitrification–denitrification in their root zone and influencing the direction of N_2_O flux (Sun et al., 2016). Kim, Kim (Kim et al., 2021) found that actively growing rice plants reduced net N_2_O emissions compared to unplanted controls, likely by facilitating complete denitrification to N_2_ or absorbing N_2_O in the soil-plant system. Such observations underscore that root traits (like gas conductive tissue, root length density, etc.) modulate the microenvironment for N-cycling microbes.

### 5.4 Impacts of nitrogen-fixing symbioses on plant-associated N_2_O fluxes

Plant symbionts also significantly alter N_2_O dynamics. Some leguminous plants, including lucerne and white clover, establish a symbiotic relationship with N-fixing rhizobium bacteria. These bacteria take dinitrogen (N_2_) present in the atmosphere and convert it into NH_4_^+^, which then can go through a process of nitrification or and subsequent denitrification. The N_2_O molecule is formed as a byproduct during nitrification and is an obligate intermediary in denitrification (Ansari et al., 2023). The decomposition of plant litter under anaerobic conditions can also increase N_2_O emissions, because microbial activity facilitates denitrification (Khokhar and Park, 2021). Arbuscular mycorrhizal endophytic (AM) fungi, which form mutualistic associations with most land plants, are a prime example. The AM fungi can enhance plant uptake of nutrients (including N), effectively sequestering N in plant biomass that might otherwise fuel N_2_O-producing processes (Bender et al., 2014). Moreover, AM fungal hyphae influence soil microbial competition and carbon distribution. Soils with active AM fungi communities emit considerably less N_2_O than soils without AM fungi. For instance, Bender, Plantenga (Bender et al., 2014) observed that disrupting AM fungi symbiosis led to a 33–42% increase in N_2_O emissions in controlled experiments. The presence of AM fungi was correlated with lower abundance of *nirK*-type denitrifiers and higher abundance of nosZ-containing microbes (N_2_O consumers) in the soil. It has also been reported that AM fungi reduce N_2_O emissions as a result of their ability to outcompete slow-growing nitrifiers for NH_4_^+^ (Storer et al., 2018; Li et al., 2023). Soils with AM fungi had lower levels of NO_3_^-^ and NH_4_^+^, and the presence of these fungi reduced the activity of bacteria responsible for NH_3_ oxidation and denitrification (Li et al., 2023). This suggests that plants with mycorrhizal fungi supply more nutrients to their fungal partners, leaving less available for soil microbes that produce N_2_O, which helps reduce emissions. Other plant-microbe symbioses can similarly modulate N_2_O: for example, legume–rhizobium symbiosis can either exacerbate or mitigate N_2_O depending on the rhizobia strain’s denitrification capabilities (Itakura et al., 2013). Microbes living inside plants and the fungi around them can emit N_2_O if they lack the enzyme to remove it, unless the plant controls O_2_ and carbon levels in its tissues (Ma et al., 2008). From a plant trait perspective, those plants that maintain symbiotic associations (mycorrhizal, N-fixers) or that produce N-cycle-targeted metabolites effectively act as active modulators of the N cycle.

The emerging picture is that N_2_O emissions are not solely a function of soil chemistry or microbial potential, but are strongly governed by the living, dynamic interface between plant roots, their microbiomes, and the soil, an interface which can be leveraged to help balance agricultural productivity and climate impacts.

Plant–rhizosphere interactions strongly shape soil nitrogen transformations and, consequently, the pool of N_2_O available for plant uptake, transport, or release to the atmosphere. However, most evidence indicates that these effects primarily operate through modifications of microbial nitrification and denitrification rather than direct regulation of plant-internal N_2_O production (Subbarao et al., 2007; Subbarao et al., 2009; Coskun et al., 2017). While many plant traits and root exudates influence microbial N_2_O production in soils, their effects on plant-associated N_2_O fluxes are largely indirect and context-dependent. Distinguishing rhizosphere-driven changes in soil N_2_O production from true plant-derived or plant-associated emissions remains a key challenge for interpreting field measurements.

## 6 Endophytes and their impact on N_2_O emissions

An *endophyte* is an organism, typically a bacterium or fungus, that resides within a plant for at least a part of its life cycle without causing apparent disease (Rodriguez et al., 2009). The intricate dynamics of symbiotic associations between endophytes and plants have long been of interest (Bibi et al., 2020). Endophytes live symbiotically in various plant tissues (Khare et al., 2018), assisting plants in increasing nutrient supply (Paungfoo-Lonhienne et al., 2010; White et al., 2012), defending against insects and pathogens (Gond et al., 2015; Soares et al., 2016; Verma et al., 2018), increasing tolerance to stress (Redman et al., 2002), and modulating plant development (Verma et al., 2017; Irizarry and White, 2018).

### 6.1 *Epichloë* endophytes

The fungal endophytes *Epichloë* have a symbiotic relationship with grasses, especially those from temperate regions. It is believed that 30% of temperate grasses have a symbiotic relationship with *Epichloë* species (Leuchtmann, 1993). *Epichloë* endophytes, through their synergistic association with grasses, can impact N_2_O emissions, particularly in cool climates, decreasing N_2_O emissions during the N cycle through plant-soil interactions (Lee et al., 2021). Chen, White (Chen et al., 2022) studied *Epichloë* endophyte effects on soil microbes in low-N soils containing perennial ryegrass and reported that in their presence, soil N_2_O production was significantly reduced, mainly due to a reduction of the *nirK* gene. *Epichloë* endophytes were shown to modify N formation within the host plant (tall fescue) by affecting its absorption, movement, and utilization (Malinowski and Belesky, 1999). Altered root N-uptake and exudation affect soil N cycling, potentially increasing substrate availability (e.g., NH_4_^+^ → NO_3_^-^) for nitrification and denitrification processes known to produce N_2_O.

*Epichloë-* infected plants can lower N_2_O emissions in farm systems by changing how plants use N and modifying microorganism communities around roots (Chen et al., 2021). It has been suggested that *Epichloë* endophytes probably increase the plant’s ability to compete with nitrifying and denitrifying bacteria for soil N (Chen et al., 2022). Since N affects both plant growth and N_2_O emissions, competition for soil N between plants and microbes could suppress N_2_O production until the plants’ N requirements are met. Furthermore, if plants outcompete N_2_O-producing microbes for soil N, N_2_O emissions are likely to remain low; however, once plant demand for N declines, N_2_O production may increase due to the greater availability of N for microbial activity (McSwiney and Robertson, 2005). Jin, Bowatte (Jin et al., 2019) investigated the impact of the endophyte *Epichloë bromicola* on the chemical and microbiological properties of soil in a barley crop. They reported that *E. bromicola* enhanced barley biomass while also increasing total soil carbon and the population of AOB.

An enhanced AOB abundance suggests a combined amplification of both soil N_2_O production (via enhanced nitrification) and the plant conduit pathway for N_2_O transport. Larger plants with greater sap flow can import more soil-derived N_2_O into their vascular systems, potentially releasing it through stems or foliage via the xylem-transpiration stream (Machacova et al., 2019).

### 6.2 Bacterial endophytes

Bacterial endophytes have been shown to actively contribute to N_2_O production in plants via their denitrification capabilities. These endophytes often carry genes like *norB* that enable the reduction of NO to N_2_O, a step that plants alone cannot perform (del Rıío et al., 2004). For instance, Sun, Li (Sun et al., 2018) studied endophytic bacteria in soybean and found that, unlike *Epichloë*, the bacterial endophytes can help plants break down NO and increase N_2_O production in plants with the help of the *norB* gene. Soybean seedlings with a natural endophytic microbiome emitted significantly more N_2_O than aseptic (microbe-free) seedlings, correlating with a higher abundance of bacterial *norB* genes in the plant tissue. In that case, at least 11 genera of endophytic denitrifying bacteria were detected inside soybean plants. This suggests a symbiotic denitrification mechanism whereby endophyte-bearing plants produce N_2_O in tandem with their microbes: the plant provides NO_3_^-^ or NO_2_^-^ and carbon (photosynthates) as substrates, while the bacteria complete the conversion of NO to N_2_O (Goshima et al., 1999). Although plants can reduce NO_2_^-^ to NO, they appear to lack the enzymatic machinery to convert NO further into N_2_O. This is primarily because plants do not possess the NO reductase enzyme, or its corresponding gene, required for this conversion, as shown by Abdel-Banat, Adam (Abdel-Banat et al., 2009) who successfully introduced the fungal P450-nor gene into plant cells, effectively allowing them to produce N_2_O through this artificial pathway.

Qin, Pang (Qin et al., 2024) provided strong experimental evidence that bacterial endophytes significantly contribute to leaf N_2_O emissions. By injecting a bactericide (streptomycin) into plant branches, they observed an 81–99% reduction in *in situ* N_2_O emissions, whereas fungicide injection had no significant effect, highlighting the dominant role of bacterial endophytes. Additionally, their isolation and back-inoculation experiments with rice confirmed that specific endophytic strains could directly produce N_2_O, validating the microbial origin of these emissions within plant tissues.

### 6.3 Other fungal endophytes

In contrast to many bacterial endophytes, most *Epichloë* (as discussed above), and other fungal endophytes studied tend to mitigate or suppress N_2_O emissions. *Trichoderma asperellum*, an asymptomatic and opportunistic microbial endophytic fungus for a wide range of plants (Siddaiah et al., 2017; Poveda, 2021), and some other N_2_O-inhibiting fungi were isolated by Wu, Chen (Wu et al., 2022) and inserted into soil without plants. When *T. asperellum* was introduced to soil (in the absence of plants) at sufficient density, N_2_O emissions dropped by between 28–47% compared to control soils. This suppression was likely due to *T. asperellum* altering N transformations or microbial community dynamics in the soil (for instance, by competing for nutrients or even promoting complete denitrification to N_2_).

The available evidence indicates that endophytic microorganisms can substantially influence plant-associated N_2_O emissions and, in some systems, may dominate foliar N_2_O production. However, the prevalence and consistency of endophyte-driven N_2_O emissions across plant species, tissues, and environmental conditions remain poorly understood. Distinguishing microbial N_2_O production occurring within plant tissues from true plant-derived N_2_O production remains a major research challenge. Clarifying the role of endophytes is therefore essential for accurately attributing plant-associated N_2_O fluxes and understanding when plants act primarily as N_2_O producers, conduits, sinks, or microbial hosts.

## 7 Whole-plant transport and conduit functions

### 7.1 Conduit-mediated transport

The conduit role of plants has been confirmed in canola, barley (Chang et al., 1998), sunflower (*Helianthus annuus*) (Ferch and Römheld, 2001), rice (Yu et al., 1997), and rubber (*Ficus elastica*) (Timilsina et al., 2022b). To demonstrate this, solutions containing dissolved N_2_O were added to soil growing canola or barley (Chang et al., 1998), or using static chamber methods for sunflower (Ferch and Römheld, 2001). In all cases, plots treated with N_2_O-enriched solutions emitted more N_2_O than untreated controls, suggesting that dissolved N_2_O was taken up by roots and transported through the plant’s transpiration stream. This mechanism was further confirmed in the sunflower study, where N_2_O emissions significantly dropped at night, when stomata were closed and transpiration rates declined, highlighting the key role of stomatal regulation in N_2_O transport and emission. Yu, Wang (Yu et al., 1997) studied CH_4_ and N_2_O emissions from rice plants using static chambers and found that rice aerenchyma were responsible for transporting approximately 80% of the soil-derived CH_4_ and N_2_O emitted from the rice-soil system via gas diffusion. The study highlighted the fact that N_2_O is significantly more soluble (20×) in water than CH_4_. Conduit-mediated N_2_O transport in plants can occur through multiple physical pathways that depend on plant anatomy and habitat. In species lacking extensive aerenchyma, soil-produced N_2_O dissolves in soil water and is transported upward with the transpiration stream through the xylem, then released via stomata in leaves or lenticels in stems (Iddris et al., 2020). In contrast, wetland species such as rice and other hydrophytes possess well-developed aerenchyma that facilitates diffusive gas movement along internal gas channels, reducing resistance to gas flow and allowing soil-derived gases to move in the gas phase from roots to shoots (Ge et al., 2024; Striker, 2024; Yong et al., 2024). More generally, plant gas transport pathways involve both diffusion and sap flow, with the balance of physical mechanisms varying with species traits and environmental conditions (Ge et al., 2024; Striker, 2024). Because N_2_O is substantially more soluble in water than CH_4_, a larger fraction of soil-derived N_2_O is likely transported dissolved in xylem sap, potentially reducing the proportion released directly compared with less soluble gases that diffuse more readily through aerenchyma pathways.

Pihlatie, Ambus (Pihlatie et al., 2005) provided evidence for conduit-mediated N_2_O emissions within beech tree leaves. They found that the application of ammonium nitrate resulted in N_2_O emissions from the leaves, averaging 0.4 µg N/m² leaf area/h, which increased to 2.0 µg N/m² leaf area/h when roots were exposed to two different N_2_O concentrations (2300 and 10400 µg/l). This demonstrated that N_2_O produced in the rhizosphere can be transported via the transpiration stream and emitted from the foliage. Such findings underscore the importance of considering the canopy as a potential emission source in forest ecosystems. Lenhart, Behrendt (Lenhart et al., 2019) examined N_2_O emissions across 32 plant species using either NO_3_^-^, NH_3_, or glycine as precursors. N_2_O was only produced where NO_3_^-^ was applied. They found that plants also produced N_2_O in the dark, which contradicts the findings of Dean and Harper (1986), Hakata, Takahashi (Hakata et al., 2003), Goshima, Mukai (Goshima et al., 1999) and Smart and Bloom (2001). Lenhart, Behrendt (Lenhart et al., 2019) hypothesized that there are other ways for plants to emit N_2_O other than photo-assimilation, but they didn’t describe or discuss any mechanisms in their study. Their sterile plant experiments demonstrated a strong correlation between N_2_O emissions and respiratory CO_2_ efflux, with leaf N_2_O fluxes (4.7–151 ng N_2_O/ h/ g) closely tracking CO_2_ respiration rates (1.4–9.3 mg CO_2_/ h/ g). This consistent ratio across varying environmental conditions suggests a physiological origin of N_2_O linked to cellular metabolism. Mechanistically, this observation aligns with proposed mitochondrial pathways in which NO_2_^-^ is reduced to nitric oxide (NO), and subsequently to N_2_O, which is hypothesized to occur under hypoxic conditions (Timilsina et al., 2020b).

Considerable variation in N_2_O emissions from soils occurs due to differences in soil textures, with higher N_2_O production reported in clay soils at elevated temperatures (Cui et al., 2023): a result of the more anaerobic environment, which favors the activity of denitrifiers (Pelster et al., 2012). However, under such conditions, plants can act as conduits, transporting this microbially produced N_2_O from the soil to the atmosphere. For instance, Hakata, Takahashi (Hakata et al., 2003) studied emissions from 17 plant species and found that all of them were able to convert NO_3_^-^ to N_2_O, and in environments like clay-rich soils, the transport of soil-derived N_2_O through plants contributed significantly to observed emissions (-0.012 ± 0.20 ng N_2_O-N/g/h).

However, conduit-mediated transport appears to be species-specific. Qin, Pang (Qin et al., 2024) conducted experiments using ^15^N_2_O-labeled water in the rhizosphere to assess how much soil-derived N_2_O is transported into plants versus produced internally. They found that this treatment increased dissolved N_2_O in soil by approximately 12-fold; however, only a small fraction (5–11%) of leaf N_2_O emissions carried the ^15^N label, with ^15^N_2_O enrichment in leaves reaching just 12–15%, and this varied among species. These results demonstrate that soil-to-leaf conduit transport is species-specific and that soil-derived N_2_O contributed only a minority (5–11%) of leaf N_2_O emissions in this experimental configuration. However, low labelling could also reflect limited transfer efficiency or rapid mixing with unlabeled internal N pools, so the relative roles of internal production versus transport remain quantitatively uncertain.

Stem-mediated N_2_O emissions have also been documented. Díaz-Pinés, Heras (Díaz-Pinés et al., 2016) reported that stems of European beech and ash (*Fraxinus angustifolia*) emitted N_2_O at rates up to 18 µg N_2_O/m²/ h. These emissions varied significantly with tree species, with ash emitting more than beech, and were also influenced by stem diameter and sap flow rates. Higher emissions were associated with this increased physiological activity, suggesting that N_2_O produced in the soil or plant tissues may be transported and released through the stem as part of the transpiration stream.

Lenticels, specialized porous tissues in the periderm, have been highlighted as primary conduits for this gas exchange, allowing N_2_O to diffuse from internal stem tissues to the atmosphere (Wen et al., 2017; Yamulki and Holt, 2017; Machacova et al., 2019). Studies in boreal and temperate forests have shown that stem N_2_O fluxes follow seasonal patterns, peaking during periods of high physiological activity such as increased sap flow and cambial growth (Machacova et al., 2019; Fraser-McDonald et al., 2024). These lenticel-mediated pathways are supported by findings of higher emissions near the stem base and declining fluxes with stem height, reflecting both lenticel density and proximity to root N_2_O sources (Fraser-McDonald et al., 2024). In wetland or tropical trees, a similar mechanism operates through aerenchyma structures that connect flooded soils to stems, with lenticels again serving as key exit points for N_2_O (Rusch and Rennenberg, 1998). Altogether, stem-mediated emissions can represent up to 10 % of the total ecosystem N_2_O budget, indicating that lenticel and xylem pathways are significant but often overlooked contributors to forest N_2_O fluxes. Plants can function as effective conduits for soil-derived N_2_O, transporting dissolved gas via the xylem or, in some species, facilitating diffusive transport through aerenchyma. The efficiency of these pathways varies with plant anatomy, transpiration dynamics, and gas solubility, yet remains insufficiently quantified across species and ecosystems. As conduit-mediated transport can bypass soil-based mitigation processes, its contribution may strongly influence the timing and magnitude of observed N_2_O emissions (Pihlatie et al., 2005; Machacova et al., 2019).

### 7.2 Foliar absorption (sink) of N_2_O

In addition to acting as conduits for N_2_O transport, plants may also absorb atmospheric N_2_O through their leaves or stems. Lensi and Chalamet (1981) demonstrated that maize (*Zea mays*) seedlings absorbed up to 38% of atmospheric N_2_O introduced into sealed incubation chambers over 150 hours. This equated to approximately 15 µg N-N_2_O/seedling/h. In experiments with lower N_2_O concentrations, absorption rates were still notable, around 0.01 µg N-N_2_O/seedling/h over 50 hours. Field-based evidence further supports this sink behavior. Li, Lee (Li et al., 2011) observed negative N_2_O fluxes in cotton and soybean plants before fertilizer application, clearly indicating that these plants were absorbing N_2_O from the atmosphere rather than emitting it. This absorption capacity, both under controlled and field conditions, highlights the dual role of plants as both sources and sinks of N_2_O, though the fate of the absorbed N_2_O within plant tissues, whether stored or metabolized, remains uncertain. A possible explanation for this uptake can be that the plants may utilize atmospheric N_2_O as an alternative N source, reducing it to NH_4_^+^ via enzymatic pathways. While the exact mechanisms remain to be fully elucidated, it has been proposed that enzymes such as NR, which can catalyze the reduction of NO_3_^-^ to NO_2_^-^ may also contribute to N_2_O transformation within plant tissues (Smart and Bloom, 2001; Müller et al., 2014). Smart and Bloom (2001) suggested that N_2_O could act as a substrate in the NO_3_^-^ assimilation pathway under specific cellular conditions. Moreover, Müller, Laughlin (Müller et al., 2014) demonstrated that barley plants exposed to N_2_O showed increased N assimilation, implying a possible metabolic use of N_2_O.

The transition of plant foliage from being a net N_2_O source to a net N_2_O sink likely depends on both environmental and physiological factors. When external nitrogen availability is low, or plant nitrogen demand is high (for example, before fertilizer application in field systems), leaves can act as N_2_O sinks by absorbing N_2_O from the atmosphere (Rusch and Rennenberg, 1998; Lenhart et al., 2015). Conversely, under nitrogen-rich conditions or during periods of active internal nitrogen turnover, such as when nitrate reductase activity is high, plants more frequently function as net N_2_O sources (Smart and Bloom, 2001; Yu and Chen, 2009; Timilsina et al., 2020a).

Ambient N_2_O concentration gradients also influence the direction of foliar N_2_O exchange. Elevated atmospheric N_2_O concentrations, as occur in intensively fertilized or manure-amended systems, can promote leaf N_2_O uptake (Rusch and Rennenberg, 1998; Machacova et al., 2019), whereas in less impacted or pristine environments with lower background N_2_O concentrations, internally produced N_2_O is more likely to be emitted to the atmosphere (Lenhart et al., 2019; Qin et al., 2019).

Once N_2_O enters the leaf, its fate remains poorly understood. One proposed pathway is dissolution in cellular fluids followed by potential assimilation, with hypotheses suggesting that N_2_O could be enzymatically reduced to NH_4_^+^ and incorporated into amino acids via pathways overlapping with NO_3_^-^/NO_2_^-^ assimilation (Smart and Bloom, 2001; Müller et al., 2014). Another possibility is biological reduction by endophytic or phyllosphere microorganisms possessing N_2_O reductase, which could convert absorbed N_2_O to N_2_ within leaf tissues. However, evidence to date suggests that many characterized foliar endophytes tend to produce rather than consume N_2_O (Rusch and Rennenberg, 1998; Lenhart et al., 2015).

*In situ* measurements provide compelling evidence that foliage can function as a substantial N_2_O sink. In a mature beech forest, Machacova, Vainio (Machacova et al., 2019) demonstrated that shoots acted as significant N_2_O sinks, sufficient to dominate ecosystem-scale N_2_O exchange and offset soil-derived emissions. Because plants can both emit and absorb N_2_O, the net sign and magnitude of plant–atmosphere exchange depend on the balance between biochemical production, conduit transport, and foliar uptake; this balance has not yet been quantified across ecosystems. So, the fate of absorbed N_2_O, whether reduced, re-emitted, or incorporated into organic N, remains uncertain and warrants further investigation. Identifying the conditions under which plants switch between source and sink behavior, such as plant developmental stage, diurnal stomatal dynamics, nitrogen status, and associations with specific microbial communities, is critical for accurately assessing the net role of vegetation in regulating atmospheric N_2_O.

Current evidence supports the involvement of multiple, partially independent pathways in plant-associated N_2_O exchange, including internal biochemical processes, microbial activity within plant tissues, and transport of soil-derived N_2_O through plants. However, the biochemical mechanisms underlying plant-internal N_2_O production remain unresolved, and the relative importance of each pathway under field conditions is not yet well quantified. Resolving these uncertainties is essential for distinguishing plant versus microbial contributions and for accurately integrating plant processes into N_2_O budgets.

These data are insufficient to define universal threshold values for transitions between source and sink behavior. Factors such as nitrogen availability (Smart and Bloom, 2001), atmospheric N_2_O concentration (Lensi and Chalamet, 1981), oxygen status (Allègre et al., 2004; Timilsina et al., 2022a), plant developmental stage (Li et al., 2011; Machacova et al., 2019), and environmental conditions (Lenhart et al., 2019; Qin et al., 2020) appear to be important regulators of this balance. However, it remains unclear whether source-sink transitions represent gradual shifts in net N_2_O exchange or threshold-driven responses under specific physiological and environmental conditions.

## 8 Environmental and physiological controls on plant N_2_O emissions

Net plant-associated N_2_O flux emerges from the interaction of multiple plant traits and environmental drivers rather than from any single factor acting in isolation. Root system size and architecture influence soil nitrogen uptake and the availability of microbially produced N_2_O for plant transport, while leaf area and stomatal density regulate the capacity for N_2_O release to the atmosphere. Environmental variables such as temperature, soil moisture, and oxygen availability simultaneously modulate microbial N_2_O production in the rhizosphere and internal plant nitrogen metabolism. Consequently, plants with extensive roots, large canopies, and high transpiration rates may function as strong N_2_O conduits under warm, moist conditions, yet the same plants may act as net sinks under low nitrogen availability or elevated atmospheric N_2_O concentrations (Lenhart et al., 2015; Barneze et al., 2024).

Root length increase has been reported to enhance the supply of O_2_ for nitrification in the rhizosphere, which can cause an increase in the soil NO_3_^-^ content within the rhizosphere (Pathak, 1999; Choudhury et al., 2022). Higher NO_3_^-^ availability in the plant tissues led to higher N_2_O release from the leaves, underscoring the link between NO_3_^-^ uptake and enhanced N_2_O emission (Qin et al., 2024). Similarly, larger leaf surface area can increase transpiration rates, which promotes the movement of dissolved gases such as N_2_O through the xylem. Given the high solubility of N_2_O in water, enhanced transpiration may facilitate its transfer from internal tissues to the atmosphere (Pihlatie et al., 2005; Hansen et al., 2019). Another possible factor impacting N_2_O emissions from plants is stomatal density which controls gas exchange between the air and the plant. Stomata are the gateway for the exchange of CO_2_ and water vapor between plants and the atmosphere (Xu and Zhou, 2008). Hence, leaf stomatal density can influence N_2_O emissions. For example, Borah and Baruah (2016) reported a strong association between wheat xylem size, flag leaf stomatal density and N_2_O emissions. These findings suggest that both below ground and above ground plant traits play important roles in regulating N_2_O fluxes and must be considered when evaluating plant contributions to ecosystem emissions.

The rate of N_2_O emissions from plants has also been observed to increase with increasing temperature, likely due to enhanced enzymatic activity involved in NO_3_^-^ assimilation and respiratory NO_3_^-^ reduction, as well as increased root and microbial activity that elevates NO_3_^-^ availability for uptake (Griffis et al., 2017; Lenhart et al., 2019). Rising temperatures accelerate microbial N cycling and plant metabolism, often increasing N_2_O emissions unless counterbalanced by efficient N uptake (Griffis et al., 2017). Day length has also been found to influence plant-mediated N_2_O emissions, with N_2_O emissions from plants increasing with shorter day length (winter) and decreasing with longer day length (summer) (Smith and Böhlke, 2019; Qin et al., 2024). The possible reason for this could be that the plant metabolism is sensitive to photoperiod variation (Pyl et al., 2012). Diel variation in metabolism creates variations in O_2_ concentration in the environment (soil and air). This variation in O_2_ and other parameters (e.g. temperature, pH) then affects the rate of N processing by plants, ultimately affecting the production of N_2_O (Baulch et al., 2012). During daylight, abundant O_2_ and photosynthetic energy allow plants to assimilate NO_3_^-^ into NH_4_^+^, minimizing gaseous losses efficiently. In contrast, nighttime hypoxia, especially in roots, limits O_2_ availability and disrupts this assimilation pathway. Nitrate reductase (NR) continues to reduce NO_3_^-^ to NO_2_^-^ in the cytosol while the subsequent reduction of NO_2_^-^ to NH_4_^+^ by mitochondria is strongly suppressed, leading to NO_2_^-^ accumulation (Timilsina et al., 2020b). For example, Allègre, Silvestre (Allègre et al., 2004) found that tomato roots maintained NR activity and accumulated NO_2_^-^ under anoxic conditions, suggesting a potential adaptive function. Oliveira and Sodek (2013) observed that O_2_ deficiency in soybean roots sharply reduced NO_3_^-^ assimilation into amino acids, resulting in cytosolic NO_2_^-^ build-up. This excess NO_2_^-,^ under low-O_2_ conditions, is directly linked to N_2_O production. Isotopic tracer studies have confirmed that NO_2_^-^ serves as a substrate for N_2_O formation in plant tissues. Experimental evidence directly links nitrite accumulation within plant tissues to N_2_O production. Goshima, Mukai (Goshima et al., 1999) demonstrated that aseptically grown tobacco supplied with ¹^5^NO_2_^-^ emitted ¹^5^N-labelled N_2_O, confirming nitrite as a direct precursor for plant-derived N_2_O. In addition, nitrite reductase (NiR)-deficient transgenic tobacco accumulated elevated NO_2_^-^ concentrations and released substantially more N_2_O than wild-type plants when supplied with NO_3_^-^, while N_2_O production was suppressed by tungstate, a nitrate reductase inhibitor, confirming that NR-derived NO_2_^-^ was the source of N_2_O. By contrast, studies such as Allègre, Silvestre (Allègre et al., 2004) and Oliveira and Sodek (2013) documented substantial nitrite accumulation under hypoxic conditions without directly quantifying N_2_O fluxes, thereby defining the physiological conditions under which nitrite-driven N_2_O production is likely to occur. Taken together, these studies suggest that under O_2_ limited conditions, such as at night or in waterlogged soils, accumulated cytosolic NO_2_^-^ becomes a key precursor for N_2_O formation in plant cells (Timilsina et al., 2020b).

Soil moisture plays a dual role; moderate levels favor microbial N_2_O production, while waterlogged soils enhance plant-mediated emissions through aerenchyma structures, as seen in flooded rice systems (Yu et al., 1997). Soil structure also influences these processes; compacted and clay-rich soils promote anaerobic microsites favorable for N_2_O production, whereas well-structured soils improve gas diffusion and reduce emissions (Cui et al., 2023) and increase N_2_O emissions by the plant through the conduit. Predicting plant N_2_O exchange therefore requires an integrated framework that considers plant physiology, microbial activity, and environmental context simultaneously.

A wide range of plant traits and environmental drivers influence plant-associated N_2_O fluxes, including root architecture, leaf area, stomatal behavior, temperature, soil moisture, and oxygen availability. Although the effects of individual factors are increasingly well documented, their interactions remain poorly understood and difficult to predict. Developing an integrated framework that captures these interacting controls is therefore necessary to improve the prediction of plant N_2_O emissions across species, environments, and management regimes.

**Figure 2:**
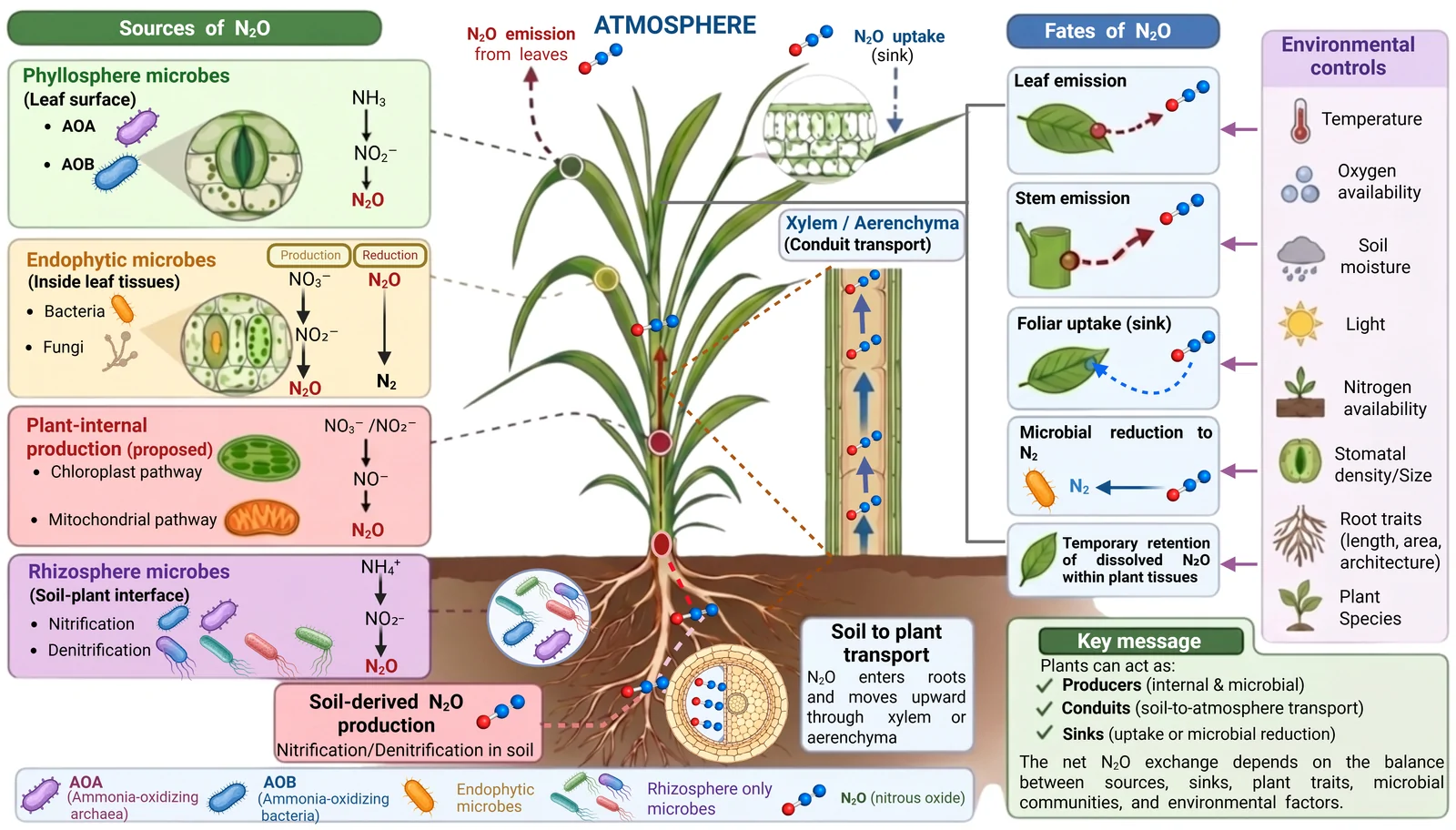
Conceptual overview of plant-associated N_2_O exchange. Plants can function as N_2_O producers, conduits, sinks, and microbial hosts, with net N_2_O exchange determined by interactions among plant traits, microbial communities, and environmental conditions. Created in https://BioRender.com.

Collectively, current evidence suggests that plant-associated N_2_O exchange is governed by interactions among plant-intrinsic pathways, microbial communities, transport processes, and environmental drivers (**Figure 2**).

## 9 Gene regulation in plant-associated N_2_O emissions

While environmental conditions regulate the magnitude of N_2_O fluxes, the underlying biological processes are ultimately controlled at the molecular level. In particular, microbial functional genes play a central role in regulating nitrification and denitrification processes associated with plant systems.

At present, there is no evidence that higher plants possess canonical microbial denitrification genes such as *nirK*/*nirS* or *norB*; instead, plant N_2_O formation appears to rely on nitrate reductase and other enzymes that are not homologous to microbial denitrification pathways. This indicates that plant-derived N_2_O production is fundamentally distinct from microbial denitrification and is instead driven by alternative enzymatic pathways, primarily involving nitrate reductase. Also, the biochemical mechanisms of N_2_O production in plants are still being unraveled; much of what is known about N_2_O emissions comes from microbial studies, where specific genes govern key enzymatic steps in nitrification and denitrification processes. Given the physiological similarities in NO_3_^-^ reduction pathways between plants and microbes, understanding these microbial genes provides a valuable framework to investigate whether analogous genetic pathways might exist in plants. The following section outlines the core microbial genes involved in N_2_O production, offering insights that could guide future research into plant genetic contributions.

The main pathways for N_2_O production by microbes are nitrification, nitrifier-denitrification, denitrification, and nitrification-coupled denitrification (Hu et al., 2015; Tian et al., 2020). With the aid of modern molecular approaches such as high-throughput sequencing and transcriptomics, various genes responsible for microbial N_2_O emissions have been identified. Five genes (*nar*G, *nirS*, *nirK*, *norB* and *nos*Z) are considered responsible for affecting the emissions of N_2_O in the denitrification process. Among these, *nirS*, *nirK*, and *nos*Z have been the focus of extensive research. In contrast, *norB* remains relatively underexplored regarding its impact on N_2_O emissions. Except for *nos*Z, there is a positive correlation between the abundance of these genes and N_2_O emissions (Yang et al., 2017). Four key genes *narG*, *nirK/nirS*, *norB*, and *nosZ* are responsible for encoding the major reductase enzymes involved in N_2_O production: nitrate reductase, nitrite reductase, nitric oxide reductase, and N_2_O reductase, respectively (Zumft, 1997). The *nar*G gene encodes NO_3_^-^ reductase and is responsible for the conversion of NO_3_^-^ to NO_2_^-,^ while *nirK* and *nirS* encode the NO_2_^-^ reductase enzyme which is responsible for conversion of NO_2_^-^ to NO; *norB* encodes the NO reductase enzyme, which is responsible for conversion of NO to N_2_O, while *nos*Z is known to be responsible for encoding the N_2_O reductase enzyme which is responsible for conversion of N_2_O to N_2_ (Hallin and Lindgren, 1999; Braker and Tiedje, 2003). The *nirK* and *nirS* genes differ structurally, but are similar in function and phylogenetics, and these two genes rarely occur in the same bacterial strains (Heylen et al., 2006).

The presence of plants has been shown to increase the abundance and activity of denitrification genes in associated microbial communities. For instance, microbial populations carrying *nirK, nirS*, and *nosZ* genes were more abundant in the rhizosphere of wheat (Hamonts et al., 2013), maize (Mahmood et al., 1997), and barley (Højberg et al., 1996) compared to unplanted soils. This is attributed to the increased carbon supply through root exudates, which provides substrates for denitrifying microbes and stimulates gene expression (Ai et al., 2020). This contrast highlights a central open question: whether plants encode any yet-unidentified NO or N_2_O reductases, or whether all steps beyond NO_2_^-^ reduction are effectively outsourced to their microbial partners.

Overall, while microbial genes involved in nitrification and denitrification provide a useful framework for understanding potential N_2_O-related pathways, direct genetic evidence for plant-specific N_2_O production remains limited. Plants possess components of nitrate and nitrite metabolism that may indirectly contribute to N_2_O formation, but no dedicated plant genes analogous to microbial N_2_O-producing enzymes have been identified. Resolving this gap is critical for distinguishing internal plant pathways from microbially mediated processes and for advancing molecular approaches to partition plant and microbial N_2_O sources.

## 10 Research gaps and future prospects

Despite increasing recognition of plants as sources of N_2_O, major gaps remain in understanding the underlying mechanisms, influencing factors, and global significance of this pathway. One key challenge is accurately distinguishing between N_2_O emitted directly by plants and that produced by associated microbial communities, especially under field conditions. While recent studies have used sterile culture systems and tracer techniques to isolate plant emissions, more work is needed to standardize methods and validate findings across different plant species and environments.

Another major gap lies in the biochemical and genetic basis of plant N_2_O production. While NR and related enzymes are implicated, the precise steps leading to N_2_O formation remain unresolved, especially under varying O_2_ or NO_3_^-^ conditions. Similarly, whether plants possess or express genes analogous to microbial denitrification genes (e.g., *nirK*, *norB*) remains an open question, and transcriptomic studies may help uncover plant-specific pathways.

The interaction between plants and their endophytic microbes is also an area of growing interest, but with limited clarity. Studies have shown that endophytic bacteria and fungi can either enhance or suppress plant N_2_O emissions, depending on the species involved and environmental conditions. However, these findings are largely limited to a few plant types, and broader comparisons across species, climates, and agricultural systems are lacking.

In addition, the role of leaf-surface microbial communities, particularly AOA and AOB, is an emerging but underexplored component of plant-related N_2_O fluxes. These microorganisms have been detected on foliage in forests and grasslands and can oxidize NH_3_ to NO_2_^-^ or release N_2_O via nitrifier-denitrification. Yet, their abundance, activity, and contribution across ecosystems are still poorly understood. Future research should examine how plant traits, canopy structure, and N availability influence the colonization and activity of phyllosphere nitrifiers.

Although certain plant traits such as high N uptake efficiency, deep rooting, or biological nitrification inhibition (BNI) have been linked to reduced N_2_O emissions, their effectiveness across genotypes and environments remains variable. Comparative studies are needed to identify which traits consistently mitigate N_2_O fluxes and how they interact with microbial partners and soil conditions.

Importantly, the potential for plants to act as sinks for atmospheric N_2_O remains largely unexplored. While several studies have demonstrated foliar N_2_O uptake under both laboratory and field conditions, the physiological mechanisms involved, such as possible reduction to NH_3_ or incorporation into organic N, are not fully understood. Additionally, the conditions under which plants switch from being sources to sinks of N_2_O are poorly defined. Addressing this gap could fundamentally change how plants are considered in global N_2_O budgets and may open new avenues for mitigation strategies.

Finally, the contribution of plant-mediated emissions to the global N_2_O budget remains difficult to quantify. Recent global estimates of plant-associated foliar N_2_O emissions (Qin et al., 2024; Tian et al., 2024) have been derived by scaling foliar emission factors from a limited number of species across global plant functional types and leaf area distributions, introducing considerable uncertainty into current assessments. Nevertheless, recognizing plants as active participants in N_2_O cycling, functioning as sources, sinks, and conduits, is essential for improving ecosystem-scale N_2_O budgets and for designing mitigation strategies that integrate plant physiology with soil microbial processes. While individual plant emission rates are often substantially lower than ecosystem-scale soil fluxes, the vast global biomass and surface area of vegetation suggest that cumulative plant-associated contributions cannot be neglected and warrant further quantification. Addressing these gaps will not only improve understanding of the plant role in global N_2_O budgets but will also support the development of mitigation strategies, such as selecting low-emission plant varieties, breeding for specific root traits, or managing beneficial microbial partnerships.

Despite increasing evidence of plant-associated N_2_O production, microbial associations, and conduit-mediated transport, soil microbial nitrification and denitrification remain the dominant sources of N_2_O in most terrestrial ecosystems. Consequently, the relative contribution of plant-associated pathways to ecosystem and global N_2_O budgets remains uncertain and requires further quantification across species, environments, and management systems.

Future research should prioritize five key areas: (i) verification of internal plant N_2_O production using axenic systems, (ii) improved isotope-tracing approaches capable of distinguishing plant-derived, microbial-derived, and soil-derived N_2_O sources, (iii) identification of the biochemical mechanisms responsible for NO-to-N_2_O conversion within plant tissues, (iv) quantification of species-specific variability under contrasting environmental conditions, and (v) integration of plant-associated processes into ecosystem- and global-scale N_2_O budgets. Addressing these priorities will be essential for reducing current uncertainties and improving representation of plant-associated N_2_O processes in global N_2_O inventories and mitigation frameworks.

## 11 Conclusion

Plants are active participants in N_2_O cycling, but several critical research priorities remain. Resolving the biochemical origin of plant-associated N_2_O is essential, particularly to distinguish internal plant production from microbial contributions. Achieving this will require integrated approaches combining isotopic tracing, sterile or gnotobiotic systems, and molecular tools capable of identifying plant-specific enzymatic pathways. In parallel, the role of plant-associated microbiomes warrants systematic evaluation. Although endophytic bacteria and phyllosphere nitrifiers have been shown to contribute to N_2_O emissions in select species, their prevalence, activity, and environmental sensitivity across plant functional types remain poorly constrained, highlighting the need for targeted manipulative experiments. Finally, scaling plant-mediated N_2_O emissions from organs to ecosystems remains a major challenge. While individual plant fluxes are often small, the global extent of vegetation suggests cumulative impacts may be substantial, underscoring the importance of incorporating plant traits and plant–microbe interactions into ecosystem and earth system models to better constrain plant contributions to global N_2_O budgets.

## References

[B1] Abalos D. De Deyn G. B. Kuyper T. W. Van Groenigen J. W. (2014a). Plant species identity surpasses species richness as a key driver of N_2_O emissions from grassland. Global Change Biol. 20, 265–275. doi: 10.1111/gcb.12350 23939815

[B2] Abalos D. Jeffery S. Sanz-Cobena A. Guardia G. Vallejo A. (2014b). Meta-analysis of the effect of urease and nitrification inhibitors on crop productivity and nitrogen use efficiency. Agric. Ecosyst. Environ. 189, 136–144. doi: 10.1016/j.agee.2014.03.036 38826717

[B3] Abdel-Banat B. Adam S. Morikawa H. (2009). Functional analysis of Fusarium oxysporum nitric oxide reductase expressed in plant suspension-cultured cells. Biotechnology 8, 204–211. doi: 10.3923/biotech.2009.204.211

[B4] Aguilar J. Rogers E. Stallman M. Windle J. Castellano M. Wright E. . (2022). Enhanced root growth reduces nitrous oxide emissions. (Authorea Preprints). doi: 10.1002/essoar.10508787.2

[B5] Ahmed Z. Gui D. Qi Z. Liu J. Ali A. Murtaza G. . (2022). “ Greenhouse gas emissions and mitigation strategies in rice production systems,” in Global Agricultural Production: Resilience to Climate Change. Ed. Ahmed M. ( Springer International Publishing, Cham), 237–265. doi: 10.1007/978-3-031-14973-3_8

[B6] Ai C. Zhang M. Sun Y. Zhang L. Zeng L. Liu Y. . (2020). Wheat rhizodeposition stimulates soil nitrous oxide emission and denitrifiers harboring the nosZ clade I gene. Soil Biol. Biochem. 143, 107738. doi: 10.1016/j.soilbio.2020.107738 38826717

[B7] Allègre A. Silvestre J. Morard P. Kallerhoff J. Pinelli E. (2004). Nitrate reductase regulation in tomato roots by exogenous nitrate: a possible role in tolerance to long-term root anoxia. J. Exp. Bot. 55, 2625–2634. doi: 10.1093/jxb/erh258 15475378

[B8] Ansari J. Udawatta R. P. Anderson S. H. (2023). Soil nitrous oxide emission from agroforestry, rowcrop, grassland and forests in North America: a review. Agrofor. Syst. 97, 1465–1479. doi: 10.1007/s10457-023-00870-y 30311153

[B9] Arp D. J. Sayavedra-Soto L. A. Hommes N. G. (2002). Molecular biology and biochemistry of ammonia oxidation by Nitrosomonas europaea. Arch. Microbiol. 178, 250–255. doi: 10.1007/s00203-002-0452-0 12209257

[B10] Arth I. Frenzel P. Conrad R. (1998). Denitrification coupled to nitrification in the rhizosphere of rice. Soil Biol. Biochem. 30, 509–515. doi: 10.1016/S0038-0717(97)00143-0

[B11] Baggs E. M. Cairns J. E. Mhlanga B. Petroli C. D. Chamberlin J. Karwat H. . (2023). Exploiting crop genotype-specific root-soil interactions to enhance agronomic efficiency. Front. Soil Sci. 3, 1125604. doi: 10.3389/fsoil.2023.1125604

[B12] Bakker P. A. Berendsen R. L. Doornbos R. F. Wintermans P. C. Pieterse C. M. (2013). The rhizosphere revisited: root microbiomics. Front. Plant Sci. 4, 165. doi: 10.3389/fpls.2013.00165 23755059 PMC3667247

[B13] Bano S. Wu Q. Yu S. Wang X. Zhang X. (2024). Soil properties drive nitrous oxide accumulation patterns by shaping denitrifying bacteriomes. Environ. Microbiome 19, 94. doi: 10.1186/s40793-024-00643-9 39568069 PMC11580698

[B14] Bardgett R. D. Mommer L. De Vries F. T. (2014). Going underground: root traits as drivers of ecosystem processes. Trends Ecol. Evol. 29, 692–699. doi: 10.1016/j.tree.2014.10.006 25459399

[B15] Barneze A. S. Petersen S. O. Eriksen J. De Deyn G. B. van Groenigen J. W. Abalos D. (2024). Belowground links between root properties of grassland species and N_2_O concentration across the topsoil profile. Soil Biol. Biochem. 196, 109498. doi: 10.1016/j.soilbio.2024.109498 38826717

[B16] Bartholomaeus A. Ahokas J. (1995). Inhibition of P-450 by aucubin: Is the biological activity of aucubin due to its glutaraldehyde-like aglycone? Toxicol. Lett. 80, 75–83. doi: 10.1016/0378-4274(95)03339-M 7482595

[B17] Bastviken D. Treat C. C. Pangala S. R. Gauci V. Enrich-Prast A. Karlson M. . (2023). The importance of plants for methane emission at the ecosystem scale. Aquat. Bot. 184, 103596. doi: 10.1016/j.aquabot.2022.103596 38826717

[B18] Baulch H. M. Dillon P. J. Maranger R. Venkiteswaran J. J. Wilson H. F. Schiff S. L. (2012). Night and day: Short-term variation in nitrogen chemistry and nitrous oxide emissions from streams. Freshw. Biol. 57, 509–525. doi: 10.1111/j.1365-2427.2011.02720.x 40046247

[B19] Bellido-Pedraza C. M. Calatrava V. Llamas A. Fernandez E. Sanz-Luque E. Galvan A. (2022). Nitrous oxide emissions from nitrite are highly dependent on nitrate reductase in the microalga chlamydomonas reinhardtii. Int. J. Mol. Sci. 23, 9412. doi: 10.3390/ijms23169412 36012676 PMC9409008

[B20] Bender S. F. Plantenga F. Neftel A. Jocher M. Oberholzer H.-R. Köhl L. . (2014). Symbiotic relationships between soil fungi and plants reduce N_2_O emissions from soil. Int. Soc. For. Microbial Ecol. (ISME) 8, 1336–1345. doi: 10.1038/ismej.2013.224 24351937 PMC4030222

[B21] Bibi S. N. Gokhan Z. Rajesh J. Mahomoodally M. F. (2020). Fungal endophytes associated with mangroves-chemistry and biopharmaceutical potential. S. Afr. J. Bot. 134, 187–212. doi: 10.1016/j.sajb.2019.12.016

[B22] Blomberg M. R. A. Ädelroth P. (2018). Mechanisms for enzymatic reduction of nitric oxide to nitrous oxide-A comparison between nitric oxide reductase and cytochrome c oxidase. Biochim. Biophys. Acta Bioenerg. 1859, 1223–1234. doi: 10.1016/j.bbabio.2018.09.368 30248312

[B23] Bo X. Zhang Z. Wang J. Guo S. Li Z. Lin H. . (2023). Benefits and limitations of biochar for climate-smart agriculture: a review and case study from China. Biochar 5, 77. doi: 10.1007/s42773-023-00279-x 30311153

[B24] Borah L. Baruah K. K. (2016). Nitrous oxide emission and mitigation from wheat agriculture: association of physiological and anatomical characteristics of wheat genotypes. Environ. Sci. pollut. Res. 23, 709–721. doi: 10.1007/s11356-015-5299-4 26335526

[B25] Bowatte S. Newton P. Lieffering M. Theobald P. Brock S. (2012). Plant canopy nitrous oxide emissions. (Wellington: Ministry for Primary Industries). Available online at: https://ndhadeliver.natlib.govt.nz/delivery/DeliveryManagerServlet?dps_pid=IE18264646.

[B26] Bowatte S. Newton P. C. Brock S. Theobald P. Luo D. (2015). Bacteria on leaves: a previously unrecognised source of N_2_O in grazed pastures. ISME J. 9, 265–267. doi: 10.1038/ismej.2014.118 25012902 PMC4274428

[B27] Braker G. Tiedje J. M. (2003). Nitric oxide reductase (norB) genes from pure cultures and environmental samples. Appl. Environ. Microbiol. 69, 3476–3483. doi: 10.1128/aem.69.6.3476-3483.2003 12788753 PMC161466

[B28] Brandão A. D. Sodek L. (2009). Nitrate uptake and metabolism by roots of soybean plants under oxygen deficiency. Braz. J. Plant Physiol. 21, 13–23. doi: 10.1590/S1677-04202009000100003

[B29] Butterbach-Bahl K. Baggs E. M. Dannenmann M. Kiese R. Zechmeister-Boltenstern S. (2013). Nitrous oxide emissions from soils: how well do we understand the processes and their controls? Philos. Trans. R. Soc. B. Biol. Sci. 368, 20130122. doi: 10.1098/rstb.2013.0122 23713120 PMC3682742

[B30] Chang C. Janzen H. Nakonechny E. Cho C. (1998). Nitrous oxide emission through plants. Soil Sci. Soc Am. J. 62, 35–38. doi: 10.2136/sssaj1998.03615995006200010005x

[B31] Chen X. Boeckx P. Shen S. Van Cleemput O. (1999). Emission of N_2_O from rye grass (Lolium perenne L.). Biol. Fertil. Soils 28, 393–396. doi: 10.1007/s003740050510

[B32] Chen Z. Jin Y. Yao X. Wei X. Li X. Li C. . (2021). Gene analysis reveals that leaf litter from Epichloë endophyte-infected perennial ryegrass alters diversity and abundance of soil microbes involved in nitrification and denitrification. Soil Biol. Biochem. 154, 108123. doi: 10.1016/j.soilbio.2020.108123 38826717

[B33] Chen Z. White J. F. Malik K. Chen H. Jin Y. Yao X. . (2022). Soil nutrient dynamics relate to Epichloë endophyte mutualism and nitrogen turnover in a low nitrogen environment. Soil Biol. Biochem. 174, 108832. doi: 10.1016/j.soilbio.2022.108832 38826717

[B34] Choudhury M. Hallin S. Ecke F. Hubalek V. Juhanson J. Frainer A. . (2022). Disentangling the roles of plant functional diversity and plaint traits in regulating plant nitrogen accumulation and denitrification in freshwaters. Funct. Ecol. 36, 921–932. doi: 10.1111/1365-2435.14001 40046247

[B35] Collman J. P. Devaraj N. K. Decréau R. A. Yang Y. Yan Y. L. Ebina W. . (2007). A cytochrome c oxidase model catalyzes oxygen to water reduction under rate-limiting electron flux. Science 315, 1565–1568. doi: 10.1126/science.1135844 17363671 PMC3064436

[B36] Coskun D. Britto D. T. Shi W. Kronzucker H. J. (2017). How plant root exudates shape the nitrogen cycle. Trends Plant Sci. 22, 661–673. doi: 10.1016/j.tplants.2017.05.004 28601419

[B37] Cui P. Chen Z. Fan F. Yin C. Song A. Li T. . (2023). Soil texture is an easily overlooked factor affecting the temperature sensitivity of N_2_O emissions. Sci. Total Environ. 862, 160648. doi: 10.1016/j.scitotenv.2022.160648 36502980

[B38] Dean J. V. Harper J. E. (1986). Nitric oxide and nitrous oxide production by soybean and winged bean during the *in vivo* nitrate reductase assay. Plant Physiol. 82, 718–723. doi: 10.1104/pp.82.3.718 16665099 PMC1056196

[B39] de Klein C. A. Harvey M. J. Clough T. J. Petersen S. O. Chadwick D. R. Venterea R. T. (2020). Global Research Alliance N_2_O chamber methodology guidelines: Introduction, with health and safety considerations. J. Environ. Qual. 49, 1073–1080. doi: 10.1002/jeq2.20118 33016437

[B40] del Rıío L. A. Javier Corpas F. Barroso J. B. (2004). Nitric oxide and nitric oxide synthase activity in plants. Phytochemistry 65, 783–792. doi: 10.1016/j.phytochem.2004.02.001 15081277

[B41] Díaz-Pinés E. Heras P. Gasche R. Rubio A. Rennenberg H. Butterbach-Bahl K. . (2016). Nitrous oxide emissions from stems of ash (Fraxinus angustifolia Vahl) and European beech (Fagus sylvatica L.). Plant Soil 398, 35–45. doi: 10.1007/s11104-015-2629-8

[B42] Ding K. Luo J. Welten B. de Klein C. A. M. (2025). The effect of plantain (Plantago lanceolata L.) in pasture swards on gaseous nitrogen emissions from nitrogen-enriched urine patches. Plant Soil 509, 363–376. doi: 10.1007/s11104-024-06865-7 30311153

[B43] Elrys A. S. Desoky E.-S. Abo El-Maati M. F. Elnahal A. S. Abdo A. I. Raza S. . (2019). Can secondary metabolites extracted from moringa seeds suppress ammonia oxidizers to increase nitrogen use efficiency and reduce nitrate contamination in potato tubers? Ecotoxicology Environ. Saf. 185, 109689. doi: 10.1016/j.ecoenv.2019.109689 31550566

[B44] Favela A. Bohn M. O. Kent A. D. (2023). Application of plant extended phenotypes to manage the agricultural microbiome belowground. Front. Microbiomes 2, 1157681. doi: 10.3389/frmbi.2023.1157681 41853376 PMC12993543

[B45] Ferch N. J. Römheld V. (2001). “ Release of water-dissolved nitrous oxide by plants: Does the transpiration water flow contribute to the emission of dissolved N_2_O by sunflower?,” in Plant Nutrition: Food Security and Sustainability of Agro-Ecosystems Through Basic and Applied Research. Eds. Horst W. J. Schenk M. K. Bürkert A. Claassen N. Flessa H. Frommer W. B. ( Springer Netherlands), 228–229. doi: 10.1007/0-306-47624-X_110

[B46] Finzi A. C. Abramoff R. Z. Spiller K. S. Brzostek E. R. Darby B. A. Kramer M. A. . (2015). Rhizosphere processes are quantitatively important components of terrestrial carbon and nutrient cycles. Global Change Biol. 21, 2082–2094. doi: 10.1111/gcb.12816 25421798

[B47] Florio A. Pommier T. Gervaix J. Bérard A. Le Roux X. (2017). Soil C and N statuses determine the effect of maize inoculation by plant growth-promoting rhizobacteria on nitrifying and denitrifying communities. Sci. Rep. 7, 8411. doi: 10.1038/s41598-017-08589-4 28827706 PMC5566440

[B48] Fowler D. Coyle M. Skiba U. Sutton M. A. Cape J. N. Reis S. . (2013). The global nitrogen cycle in the twenty-first century. Philos. Trans. R. Soc. B. Biol. Sci. 368, 20130164. doi: 10.1098/rstb.2013.0164 23713126 PMC3682748

[B49] Fraser-McDonald A. Boardman C. Gladding T. Burnley S. Gauci V. (2024). Nitrous oxide (N2O) emissions from a forested closed landfill site. (Vienna: EGU General Assembly Conference Abstracts). Available online at: https://ui.adsabs.harvard.edu/abs/2024EGUGA.26.1443F.

[B50] Gal M. Preston G. M. Massey R. C. Spiers A. J. Rainey P. B. (2003). Genes encoding a cellulosic polymer contribute toward the ecological success of Pseudomonas fluorescens SBW25 on plant surfaces. Mol. Ecol. 12, 3109–3121. doi: 10.1046/j.1365-294x.2003.01953.x 14629390

[B51] Ge M. Korrensalo A. Laiho R. Kohl L. Lohila A. Pihlatie M. . (2024). Plant-mediated CH_4_ exchange in wetlands: A review of mechanisms and measurement methods with implications for modelling. Sci. Total Environ. 914, 169662. doi: 10.1016/j.scitotenv.2023.169662 38159777

[B52] Gogoi B. Baruah K. K. (2012). Nitrous oxide emissions from fields with different wheat and rice varieties. Pedosphere 22, 112–121. doi: 10.1016/S1002-0160(11)60197-5 29786478

[B53] Gond S. K. Bergen M. S. Torres M. S. White J. F. Kharwar R. N. (2015). Effect of bacterial endophyte on expression of defense genes in Indian popcorn against Fusarium moniliforme. Symbiosis 66, 133–140. doi: 10.1007/s13199-015-0348-9 30311153

[B54] Goshima N. Mukai T. Suemori M. Takahashi M. Caboche M. Morikawa H. (1999). Emission of nitrous oxide (N_2_O) from transgenic tobacco expressing antisense NiR mRNA. Plant J. 19, 75–80. doi: 10.1046/j.1365-313x.1999.00494.x 10417728

[B55] Graf D. R. Jones C. M. Hallin S. (2014). Intergenomic comparisons highlight modularity of the denitrification pathway and underpin the importance of community structure for N_2_O emissions. PloS One 9, e114118. doi: 10.1371/journal.pone.0114118 25436772 PMC4250227

[B56] Graf D. R. H. Jones C. M. Zhao M. Hallin S. (2022). Assembly of root-associated N_2_O-reducing communities of annual crops is governed by selection for nosZ clade I over clade II. FEMS Microbiol. Ecol. 98. doi: 10.1093/femsec/fiac092 35927461 PMC9397574

[B57] Griffis T. J. Chen Z. Baker J. M. Wood J. D. Millet D. B. Lee X. . (2017). Nitrous oxide emissions are enhanced in a warmer and wetter world. Proc. Natl. Acad. Sci. 114, 12081–12085. doi: 10.1073/pnas.1704552114 29078277 PMC5692531

[B58] Gupta K. J. Lee C. P. Ratcliffe R. G. (2016). Nitrite protects mitochondrial structure and function under hypoxia. Plant Cell Physiol. 58, 175–183. doi: 10.1093/pcp/pcw174 28007968

[B59] Hakata M. Takahashi M. Zumft W. Sakamoto A. Morikawa H. (2003). Conversion of the nitrate nitrogen and nitrogen dioxide to nitrous oxides in plants. Acta Biotechnol. 23, 249–257. doi: 10.1002/abio.200390032 41531421

[B60] Hallin S. Lindgren P. E. (1999). PCR detection of genes encoding nitrite reductase in denitrifying bacteria. Appl. Environ. Microbiol. 65, 1652–1657. doi: 10.1128/aem.65.4.1652-1657.1999 10103263 PMC91233

[B61] Hamonts K. Clough T. J. Stewart A. Clinton P. W. Richardson A. E. Wakelin S. A. . (2013). Effect of nitrogen and waterlogging on denitrifier gene abundance, community structure and activity in the rhizosphere of wheat. FEMS Microbiol. Ecol. 83, 568–584. doi: 10.1111/1574-6941.12015 23006139

[B62] Hansen S. Berland Frøseth R. Stenberg M. Stalenga J. Olesen J. E. Krauss M. . (2019). Reviews and syntheses: Review of causes and sources of N_2_O emissions and NO_3_ leaching from organic arable crop rotations. Biogeosciences 16, 2795–2819. doi: 10.5194/bg-16-2795-2019

[B63] Harter J. Guzman-Bustamante I. Kuehfuss S. Ruser R. Well R. Spott O. . (2016). Gas entrapment and microbial N_2_O reduction reduce N_2_O emissions from a biochar-amended sandy clay loam soil. Sci. Rep. 6, 39574. doi: 10.1038/srep39574 28008997 PMC5180216

[B64] Henry S. Texier S. Hallet S. Bru D. Dambreville C. Chèneby D. . (2008). Disentangling the rhizosphere effect on nitrate reducers and denitrifiers: insight into the role of root exudates. Environ. Microbiol. 10, 3082–3092. doi: 10.1111/j.1462-2920.2008.01599.x 18393993

[B65] Herbert J. M. Maffrand J. P. Taoubi K. Augereau J. M. Fouraste I. Gleye J. (1991). Verbascoside isolated from Lantana camara, an inhibitor of protein kinase C. J. Nat. Prod. 54, 1595–1600. doi: 10.1021/np50078a016 1812212

[B66] Heylen K. Gevers D. Vanparys B. Wittebolle L. Geets J. Boon N. . (2006). The incidence of nirS and nirK and their genetic heterogeneity in cultivated denitrifiers. Environ. Microbiol. 8, 2012–2021. doi: 10.1111/j.1462-2920.2006.01081.x 17014499

[B67] Hockstad L. Hanel L. (2018). Inventory of US Greenhouse Gas Emissions and Sinks (United States: Environmental System Science Data Infrastructure for a Virtual Ecosystem (ESS-DIVE)(United States). Available online at: https://www.osti.gov/biblio/1464240.

[B68] Højberg O. Binnerup S. J. Sørensen J. (1996). Potential rates of ammonium oxidation, nitrite oxidation, nitrate reduction and denitrification in the young barley rhizosphere. Soil Biol. Biochem. 28, 47–54. doi: 10.1016/0038-0717(95)00119-0

[B69] Hu H.-W. Chen D. He J.-Z. (2015). Microbial regulation of terrestrial nitrous oxide formation: understanding the biological pathways for prediction of emission rates. FEMS Microbiol. Rev. 39, 729–749. doi: 10.1093/femsre/fuv021 25934121

[B70] Iddris N.-A. Corre M. D. Yemefack M. van Straaten O. Veldkamp E. (2020). Stem and soil nitrous oxide fluxes from rainforest and cacao agroforest on highly weathered soils in the Congo Basin. Biogeosciences 17, 5377–5397. doi: 10.5194/bg-17-5377-2020

[B71] IPCC (2023). Climate Change 2023: Synthesis Report. Contribution of Working Groups I, II and III to the Sixth Assessment Report of the Intergovernmental Panel on Climate Change (Geneva: World Meteorological Organization). doi: 10.59327/IPCC/AR6-9789291691647

[B72] Irizarry I. White J. F. (2018). Bacillus amyloliquefaciens alters gene expression, ROS production and lignin synthesis in cotton seedling roots. J. Appl. Microbiol. 124, 1589–1603. doi: 10.1111/jam.13744 29473989

[B73] Itakura M. Uchida Y. Akiyama H. Hoshino Y. T. Shimomura Y. Morimoto S. . (2013). Mitigation of nitrous oxide emissions from soils by Bradyrhizobium japonicum inoculation. Nat. Clim. Change 3, 208–212. doi: 10.1038/nclimate1734 37880705

[B74] Jin Y. Bowatte S. Jia Q. Hou F. Li C. (2019). Effects of Epichloë endophytic fungi infection in wild barley (Hordeum brevisubulatum) on soil chemical properties and the soil microbial community. Acta Prataculturae Sin. 28, 66–77.

[B75] Kelley L. A. Zhang Z. Tamagno S. Lundy M. E. Mitchell J. P. Gaudin A. C. M. . (2024). Changes in soil N_2_O emissions and nitrogen use efficiency following long-term soil carbon storage: Evidence from a mesocosm experiment. Agric. Ecosyst. Environ. 370, 109054. doi: 10.1016/j.agee.2024.109054 42348292

[B76] Khare E. Mishra J. Arora N. K. (2018). Multifaceted interactions between endophytes and plant: Developments and prospects. Front. Microbiol. 9, 2732. doi: 10.3389/fmicb.2018.02732 30498482 PMC6249440

[B77] Khokhar N. H. Park J.-W. (2021). Contribution of different quantities of leaf litter to nitrous oxide emission from a temperate deciduous forest. KSCE J. Civ. Eng. 25, 1163–1175. doi: 10.1007/s12205-021-1441-7 30311153

[B78] Kim G. W. Kim P. J. Khan M. I. Lee S.-J. (2021). Effect of rice planting on nitrous oxide (N_2_O) emission under different levels of nitrogen fertilization. Agronomy 11. doi: 10.3390/agronomy11020217 30654563

[B79] Klepper L. (1979). Nitric oxide (NO) and nitrogen dioxide (NO_2_) emissions from herbicide-treated soybean plants. Atmos. Environ. 13, 537–542. doi: 10.1016/0004-6981(79)90148-3

[B80] Lata J. C. Guillaume K. Degrange V. Abbadie L. Lensi R. (2000). Relationships between root density of the African grass Hyparrhenia diplandra and nitrification at the decimetric scale: an inhibition-stimulation balance hypothesis. Proc. R. Soc. B. Biol. Sci. 267, 595–600. doi: 10.1098/rspb.2000.1043 10787164 PMC1690563

[B81] Lee K. Missaoui A. Mahmud K. Presley H. Lonnee M. (2021). Interaction between grasses and Epichloë endophytes and its significance to biotic and abiotic stress tolerance and the rhizosphere. Microorganisms 9, 2186. doi: 10.3390/microorganisms9112186 34835312 PMC8623577

[B82] Lenhart K. Behrendt T. Greiner S. Steinkamp J. Well R. Giesemann A. . (2019). Nitrous oxide effluxes from plants as a potentially important source to the atmosphere. New Phytol. 221, 1398–1408. doi: 10.1111/nph.15455 30303249

[B83] Lenhart K. Weber B. Elbert W. Steinkamp J. Clough T. Crutzen P. . (2015). Nitrous oxide and methane emissions from cryptogamic covers. Global Change Biol. 21, 3889–3900. doi: 10.1111/gcb.12995 26152454

[B84] Lensi R. Chalamet A. (1981). Absorption de l'oxyde nitreux par les parties aeriennes du maïs. Plant Soil 59, 91–98. doi: 10.1007/bf02183595 30311153

[B85] Leuchtmann A. (1993). Systematics, distribution, and host specificity of grass endophytes. Nat. Toxins 1, 150–162. doi: 10.1002/nt.2620010303 1344916

[B86] Li X. He G. Li D. Bei S. Luan D. Sun X. . (2023). Arbuscular mycorrhizal fungi reduce N_2_O emissions from degraded residue patches. Front. Ecol. Evol. 11, 1224849. doi: 10.3389/fevo.2023.1224849

[B87] Li J. Lee X. Yu Q. Tong X. Qin Z. Macdonald B. (2011). Contributions of agricultural plants and soils to N_2_O emission in a farmland. Biogeosciences Discuss. 2011, 5505–5535. doi: 10.5194/bgd-8-5505-2011

[B88] Lifeng Z. Boeckx P. Guanxiong C. Van Cleemput O. (2000). Nitrous oxide emission from herbicide-treated soybean. Biol. Fertil. Soils 32, 173–176. doi: 10.1007/s003740000235 30311153

[B89] Luo J. Balvert S. F. Wise B. Welten B. Ledgard S. F. de Klein C. A. M. . (2018). Using alternative forage species to reduce emissions of the greenhouse gas nitrous oxide from cattle urine deposited onto soil. Sci. Total Environ. 610-611, 1271–1280. doi: 10.1016/j.scitotenv.2017.08.186 28851147

[B90] Ma W. Farrell R. Siciliano S. (2008). Soil formate regulates the fungal nitrous oxide emission pathway. Appl. Environ. Microbiol. 74, 6690–6696. doi: 10.1128/aem.00797-08 18791019 PMC2576722

[B91] Machacova K. Vainio E. Urban O. Pihlatie M. (2019). Seasonal dynamics of stem N_2_O exchange follow the physiological activity of boreal trees. Nat. Commun. 10, 4989. doi: 10.1038/s41467-019-12976-y 31676776 PMC6825224

[B92] Mahmood T. Ali R. Malik K. A. Shamsi S. R. A. (1997). Denitrification with and without maize plants (Zea mays L.) under irrigated field conditions. Biol. Fertil. Soils 24, 323–328. doi: 10.1007/s003740050251 30311153

[B93] Malinowski D. P. Belesky D. P. (1999). Neotyphodium coenophialum-endophyte infection affects the ability of tall fescue to use sparingly available phosphorus. J. Plant Nutr. 22, 835–853. doi: 10.1080/01904169909365675

[B94] Mander Ü. Krasnova A. Escuer-Gatius J. Espenberg M. Schindler T. Machacova K. . (2021). Forest canopy mitigates soil N_2_O emission during hot moments. NPJ Clim. Atmos. Sci. 4, 39. doi: 10.1038/s41612-021-00194-7 37880705

[B95] McSwiney C. P. Robertson G. P. (2005). Nonlinear response of N_2_O flux to incremental fertilizer addition in a continuous maize (Zea mays L.) cropping system. Global Change Biol. 11, 1712–1719. doi: 10.1111/j.1365-2486.2005.01040.x 40046247

[B96] Miflin B. J. (1974). The location of nitrite reductase and other enzymes related to amino acid biosynthesis in the plastids of root and leaves. Plant Physiol. 54, 550–555. doi: 10.1104/pp.54.4.550 16658926 PMC367451

[B97] Millar N. Urrea A. Kahmark K. Shcherbak I. Robertson G. P. Ortiz-Monasterio I. (2018). Nitrous oxide (N_2_O) flux responds exponentially to nitrogen fertilizer in irrigated wheat in the Yaqui Valley, Mexico. Agric. Ecosyst. Environ. 261, 125–132. doi: 10.1016/j.agee.2018.04.003 24927583

[B98] Morikawa H. Higaki A. Nohno M. Takahashi M. Kamada M. Nakata M. . (1998). More than a 600-fold variation in nitrogen dioxide assimilation among 217 plant taxa. Plant Cell Environ. 21, 180–190. doi: 10.1046/j.1365-3040.1998.00255.x 37945311

[B99] Morris C. E. Kinkel L. L. (2002). “ Fifty years of phyllosphere microbiology: significant contributions to research in related fields,” in Phyllosphere Microbiology, eds Lindow S. E. Hecht-Poinar E. I. Elliott V. J. , ( APS Press),365–375.

[B100] Müller C. Laughlin R. J. Spott O. Rütting T. (2014). Quantification of N_2_O emission pathways via a ^15^N tracing model. Soil Biol. Biochem. 72, 44–54. doi: 10.1016/j.soilbio.2014.01.013 2050173

[B101] Nardi P. Akutsu M. Pariasca-Tanaka J. Wissuwa M. (2013). Effect of methyl 3-4 hydroxyphenyl propionate, a sorghum root exudate, on N dynamic, potential nitrification activity and abundance of ammonia-oxidizing bacteria and archaea. Plant Soil 367, 627–637. doi: 10.1007/s11104-012-1494-y 30311153

[B102] Nogueira C. B. Menéndez E. Ramírez-Bahena M. H. Velázquez E. Peix Á. Mateos P. F. . (2019). The N-fixing legume periandra mediterranea constrains the invasion of an exotic grass (Melinis minutiflora P. Beauv) by altering soil N cycling. Sci. Rep. 9, 11033. doi: 10.1038/s41598-019-47380-5 31363104 PMC6667476

[B103] Oliveira H. C. Sodek L. (2013). Effect of oxygen deficiency on nitrogen assimilation and amino acid metabolism of soybean root segments. Amino Acids 44, 743–755. doi: 10.1007/s00726-012-1399-3 22990842

[B104] Pan S.-Y. He K.-H. Lin K.-T. Fan C. Chang C.-T. (2022). Addressing nitrogenous gases from croplands toward low-emission agriculture. NPJ Clim. Atmos. Sci. 5, 43. doi: 10.1038/s41612-022-00265-3 37880705

[B105] Papen H. Gessler A. Zumbusch E. Rennenberg H. (2002). Chemolithoautotrophic nitrifiers in the phyllosphere of a spruce ecosystem receiving high atmospheric nitrogen input. Curr. Microbiol. 44, 56–60. doi: 10.1007/s00284-001-0074-9 11727042

[B106] Pathak H. (1999). Emissions of nitrous oxide from soil. Curr. Sci. 77, 359–369. Available online at: http://www.jstor.org/stable/24102954. 15571746

[B107] Paungfoo-Lonhienne C. Rentsch D. Robatzek S. Webb R. I. Sagulenko E. Näsholm T. . (2010). Turning the table: Plants consume microbes as a source of nutrients. PloS One 5, e11915. doi: 10.1371/journal.pone.0011915 20689833 PMC2912860

[B108] Pelster D. E. Chantigny M. H. Rochette P. Angers D. A. Rieux C. Vanasse A. (2012). Nitrous oxide emissions respond differently to mineral and organic nitrogen sources in contrasting soil types. J. Environ. Qual. 41, 427–435. doi: 10.2134/jeq2011.0261 22370405

[B109] Philippot L. Hallin S. Börjesson G. Baggs E. (2009). Biochemical cycling in the rhizosphere having an impact on global change. Plant Soil 321, 61–81. doi: 10.1007/s11104-008-9796-9 30311153

[B110] Pihlatie M. Ambus P. Rinne J. Pilegaard K. Vesala T. (2005). Plant-mediated nitrous oxide emissions from beech (Fagus sylvatica) leaves. New Phytol. 168, 93–98. doi: 10.1111/j.1469-8137.2005.01542.x 16159324

[B111] Poderoso J. J. Helfenberger K. Poderoso C. (2019). The effect of nitric oxide on mitochondrial respiration. Nitric. Oxide 88, 61–72. doi: 10.1016/j.niox.2019.04.005 30999001

[B112] Poveda J. (2021). Glucosinolates profile of Arabidopsis thaliana modified root colonization of Trichoderma species. Biol. Control 155, 104522. doi: 10.1016/j.biocontrol.2020.104522 38826717

[B113] Pyl E.-T. Piques M. Ivakov A. Schulze W. Ishihara H. Stitt M. . (2012). Metabolism and growth in Arabidopsis depend on the daytime temperature but are temperature-compensated against cool nights. Plant Cell 24, 2443–2469. doi: 10.1105/tpc.112.097188 22739829 PMC3406903

[B114] Qin S. Pang Y. Hu H. Liu T. Yuan D. Clough T. . (2024). Foliar N_2_O emissions constitute a significant source to atmosphere. Global Change Biol. 30, e17181. doi: 10.1111/gcb.17181 38372171

[B115] Qin H. Xing X. Tang Y. Hou H. Yang J. Shen R. . (2019). Linking soil N_2_O emissions with soil microbial community abundance and structure related to nitrogen cycle in two acid forest soils. Plant Soil 435, 95–109. doi: 10.1007/s11104-018-3863-7 35391544

[B116] Qin H. Xing X. Tang Y. Zhu B. Wei X. Chen X. . (2020). Soil moisture and activity of nitrite- and nitrous oxide-reducing microbes enhanced nitrous oxide emissions in fallow paddy soils. Biol. Fertil. Soils 56, 53–67. doi: 10.1007/s00374-019-01403-5 30311153

[B117] Ravishankara A. R. Daniel J. S. Portmann R. W. (2009). Nitrous oxide (N_2_O): The dominant ozone-depleting substance emitted in the 21st century. Science 326, 123–125. doi: 10.1126/science.1176985 19713491

[B118] Redman R. S. Sheehan K. B. Stout R. G. Rodriguez R. J. Henson J. M. (2002). Thermotolerance generated by plant/fungal symbiosis. Science 298, 1581. doi: 10.1126/science.1072191 12446900

[B119] Rodriguez R. White J. Jr Arnold A. E. Redman R. (2009). Fungal endophytes: diversity and functional roles. New Phytol. 182, 314–330. doi: 10.1111/j.1469-8137.2009.02773.x 19236579

[B120] Rusch H. Rennenberg H. (1998). Black alder (Alnus Glutinosa (L.) Gaertn.) trees mediate methane and nitrous oxide emission from the soil to the atmosphere. Plant Soil 201, 1–7. doi: 10.1023/a:1004331521059 41886696

[B121] Saeki Y. Nakamura M. Mason M. L. T. Yano T. Shiro S. Sameshima-Saito R. . (2017). Effect of flooding and the nosZ gene in bradyrhizobia on bradyrhizobial community structure in the soil. Microbes Environments 32, 154–163. doi: 10.1264/jsme2.me16132 28592720 PMC5478539

[B122] Sameshima-Saito R. Chiba K. Minamisawa K. (2004). New method of denitrification analysis of bradyrhizobium field isolates by gas chromatographic determination of (15)N-labeled N(2). Appl. Environ. Microbiol. 70, 2886–2891. doi: 10.1128/aem.70.5.2886-2891.2004 15128547 PMC404451

[B123] Sameshima-Saito R. Chiba K. Minamisawa K. (2006). Correlation of denitrifying capability with the existence of nap, nir, nor and nos genes in diverse strains of soybean bradyrhizobia. Microbes Environments 21, 174–184. doi: 10.1264/jsme2.21.174

[B124] Sánchez C. Minamisawa K. (2019). Nitrogen cycling in soybean rhizosphere: Sources and sinks of nitrous oxide (N_2_O). Front. Microbiol. 10, 1943. doi: 10.3389/fmicb.2019.01943 31497007 PMC6712156

[B125] Saraste M. Castresana J. (1994). Cytochrome oxidase evolved by tinkering with denitrification enzymes. FEBS Lett. 341, 1–4. doi: 10.1016/0014-5793(94)80228-9 8137905

[B126] Saud S. Wang D. Fahad S. (2022). Improved nitrogen use efficiency and greenhouse gas emissions in agricultural soils as producers of biological nitrification inhibitors. Front. Plant Sci. 13, 854195. doi: 10.3389/fpls.2022.854195 35432390 PMC9011059

[B127] Schlesinger W. H. (2010). On fertilizer-induced soil carbon sequestration in China's croplands. Global Change Biol. 16, 849–850. doi: 10.1111/j.1365-2486.2009.01958.x 40046247

[B128] Shen Y. Huang J. Wang D. Sun B. Whalen J. K. Chen Y. (2024). Maize lowers the N_2_O emissions from maize/soybean intercropping. Rhizosphere 31, 100937. doi: 10.1016/j.rhisph.2024.100937 38826717

[B129] Siddaiah C. N. Satyanarayana N. R. Mudili V. Kumar Gupta V. Gurunathan S. Rangappa S. . (2017). Elicitation of resistance and associated defense responses in Trichoderma hamatum induced protection against pearl millet downy mildew pathogen. Sci. Rep. 7, 43991. doi: 10.1038/srep43991 28322224 PMC5359564

[B130] Simon P. L. de Klein C. A. M. Worth W. Rutherford A. J. Dieckow J. (2019). The efficacy of Plantago lanceolata for mitigating nitrous oxide emissions from cattle urine patches. Sci. Total Environ. 691, 430–441. doi: 10.1016/j.scitotenv.2019.07.141 31323588

[B131] Smart D. R. Bloom A. J. (2001). Wheat leaves emit nitrous oxide during nitrate assimilation. Proc. Natl. Acad. Sci. 98, 7875–7878. doi: 10.1073/pnas.131572798 11427711 PMC35435

[B132] Smith K. (1997). The potential for feedback effects induced by global warming on emissions of nitrous oxide by soils. Global Change Biol. 3, 327–338. doi: 10.1111/j.1475-2743.2004.tb00366.x 37945311

[B133] Smith R. L. Böhlke J. K. (2019). Methane and nitrous oxide temporal and spatial variability in two midwestern USA streams containing high nitrate concentrations. Sci. Total Environ. 685, 574–588. doi: 10.1016/j.scitotenv.2019.05.374 31181534

[B134] Smith K. Conen F. (2004). Impacts of land management on fluxes of trace greenhouse gases. Soil Use Manage. 20, 255–263. doi: 10.1079/sum2004238 36007395

[B135] Soares M. A. Li H.-Y. Bergen M. da Silva J. M. Kowalski K. P. White J. F. (2016). Functional role of an endophytic Bacillus amyloliquefaciens in enhancing growth and disease protection of invasive English ivy (Hedera helix L.). Plant Soil 405, 107–123. doi: 10.1007/s11104-015-2638-7 30311153

[B136] Steffens B. Geske T. Sauter M. (2011). Aerenchyma formation in the rice stem and its promotion by H_2_O_2_. New Phytol. 190, 369–378. doi: 10.1111/j.1469-8137.2010.03496.x 21039565

[B137] Storer K. Coggan A. Ineson P. Hodge A. (2018). Arbuscular mycorrhizal fungi reduce nitrous oxide emissions from N_2_O hotspots. New Phytol. 220, 1285–1295. doi: 10.1111/nph.14931 29206293 PMC6282961

[B138] Striker G. G. (2024). “ Oxygen transport and plant ventilation,” in Responses of Plants to Soil Flooding. Eds. Sakagami J.-I. Nakazono M. ( Springer Nature Singapore, Singapore), 139–156. doi: 10.1007/978-981-99-9112-9_9

[B139] Subbarao G. V. Nakahara K. Hurtado M. P. Ono H. Moreta D. E. Salcedo A. F. . (2009). Evidence for biological nitrification inhibition in Brachiaria pastures. Proc. Natl. Acad. Sci. 106, 17302–17307. doi: 10.1073/pnas.0903694106 19805171 PMC2752401

[B140] Subbarao G. V. Rondon M. Ito O. Ishikawa T. Rao I. M. Nakahara K. . (2007). Biological nitrification inhibition (BNI)-is it a widespread phenomenon? Plant Soil 294, 5–18. doi: 10.1007/s11104-006-9159-3 30311153

[B141] Sun H. Li Y. Xu H. (2018). Is endophyte-plant co-denitrification a source of nitrous oxides emission? An experimental investigation with soybean. Agronomy 8, 108. doi: 10.3390/agronomy8070108 30654563

[B142] Sun H. Zhou S. Fu Z. Chen G. Zou G. Song X. (2016). A two-year field measurement of methane and nitrous oxide fluxes from rice paddies under contrasting climate conditions. Sci. Rep. 6, 28255. doi: 10.1038/srep28255 27321231 PMC4913302

[B143] Sundin G. (2002). “ Ultraviolet radiation on leaves: its influence on microbial communities and their adaptations,” in Phyllosphere Microbiology. Eds. Lindow S. E. Hecht-Poinar E. I. Elliott V. J. ( American Phytopathological Society (APS Press, Saint Paul, Minnesota, USA), 27–41.

[B144] Tian H. Pan N. Thompson R. L. Canadell J. G. Suntharalingam P. Regnier P. . (2024). Global nitrous oxide budget (1980–2020). Earth Syst. Sci. Data 16, 2543–2604. doi: 10.5194/essd-16-2543-2024

[B145] Tian H. Xu R. Canadell J. G. Thompson R. L. Winiwarter W. Suntharalingam P. . (2020). A comprehensive quantification of global nitrous oxide sources and sinks. Nature 586, 248–256. doi: 10.1038/s41586-020-2780-0 33028999

[B146] Tiemeyer B. Freibauer A. Borraz E. A. Augustin J. Bechtold M. Beetz S. . (2020). A new methodology for organic soils in national greenhouse gas inventories: Data synthesis, derivation and application. Ecol. Indic. 109, 105838. doi: 10.1016/j.ecolind.2019.105838 38826717

[B147] Timilsina A. Bizimana F. Pandey B. Yadav R. K. P. Dong W. Hu C. (2020a). Nitrous oxide emissions from paddies: Understanding the role of rice plants. Plants (Basel) 9. doi: 10.3390/plants9020180 32024218 PMC7076488

[B148] Timilsina A. Dong W. Hasanuzzaman M. Liu B. Hu C. (2022a). Nitrate–nitrite–nitric oxide pathway: A mechanism of hypoxia and anoxia tolerance in plants. Int. J. Mol. Sci. 23, 11522. doi: 10.3390/ijms231911522 36232819 PMC9569746

[B149] Timilsina A. Oenema O. Luo J. Wang Y. Dong W. Pandey B. . (2022b). Plants are a natural source of nitrous oxide even in field conditions as explained by ^15^N site preference. Sci. Total Environ. 805, 150262. doi: 10.1016/j.scitotenv.2021.150262 34536861

[B150] Timilsina A. Zhang C. Pandey B. Bizimana F. Dong W. Hu C. (2020b). Potential pathway of nitrous oxide formation in plants. Front. Plant Sci. 11, 1177. doi: 10.3389/fpls.2020.01177 32849729 PMC7412978

[B151] UN Environment Programme (2013). Drawing Down N2O to Protect Climate and the Ozone Layer: A UNEP Synthesis Report. Available online at: https://www.unep.org/resources/report/drawing-down-n2o-protect-climate-and-ozone-layer-unep-synthesis-report.

[B152] Vandenkoornhuyse P. Quaiser A. Duhamel M. Le Van A. Dufresne A. (2015). The importance of the microbiome of the plant holobiont. New Phytol. 206, 1196–1206. doi: 10.1016/j.micres.2021.126888 25655016

[B153] Venterea R. T. Bijesh M. Dolan M. S. (2011). Fertilizer source and tillage effects on yield-scaled nitrous oxide emissions in a corn cropping system. J. Environ. Qual. 40, 1521–1531. doi: 10.2134/jeq2011.0039 21869514

[B154] Verma S. K. Kingsley K. L. Bergen M. S. Kowalski K. P. White J. F. (2018). Fungal disease prevention in seedlings of rice (Oryza sativa) and other grasses by growth-promoting seed-associated endophytic bacteria from invasive phragmites australis. Microorganisms 6. doi: 10.3390/microorganisms6010021 29518024 PMC5874635

[B155] Verma S. K. Kingsley K. Irizarry I. Bergen M. Kharwar R. N. White J. F. Jr. (2017). Seed-vectored endophytic bacteria modulate development of rice seedlings. J. Appl. Microbiol. 122, 1680–1691. doi: 10.1111/jam.13463 28375579

[B156] Wang J.-F. Huang J.-W. Cai Z.-X. Li Q.-S. Sun Y.-Y. Zhou H.-Z. . (2024). Differential nitrous oxide emission and microbiota succession in constructed wetlands induced by nitrogen forms. Environ. Int. 183, 108369. doi: 10.1016/j.envint.2023.108369 38070437

[B157] Wang Z. Li Y. Zheng W. Ji Y. Duan M. Ma L. (2023). Ammonia oxidizing archaea and bacteria respond to different manure application rates during organic vegetable cultivation in Northwest China. Sci. Rep. 13, 8064. doi: 10.1038/s41598-023-35134-3 37202434 PMC10195796

[B158] Watanabe K. Kohzu A. Suda W. Yamamura S. Takamatsu T. Takenaka A. . (2016). Microbial nitrification in throughfall of a Japanese cedar associated with archaea from the tree canopy. SpringerPlus 5, 1596. doi: 10.1186/s40064-016-3286-y 27652169 PMC5026986

[B159] Wen Y. Corre M. D. Rachow C. Chen L. Veldkamp E. (2017). Nitrous oxide emissions from stems of alder, beech and spruce in a temperate forest. Plant Soil 420, 423–434. doi: 10.1007/s11104-017-3416-5 30311153

[B160] White J. F. Crawford H. Torres M. S. Mattera R. Irizarry I. Bergen M. (2012). A proposed mechanism for nitrogen acquisition by grass seedlings through oxidation of symbiotic bacteria. Symbiosis 57, 161–171. doi: 10.1007/s13199-012-0189-8 23087539 PMC3473182

[B161] World Meteorological Organization (2022). The State of Greenhouse Gases in the Atmosphere Using Global Observations Through 2021 (Geneva: World Meteorological Organization). 2022 Contract No.: WMO Greenhouse Gas Bulletin No. 18. Available online at: https://reliefweb.int/report/world/wmo-greenhouse-gas-bulletin-ghg-bulletin-state-greenhouse-gases-atmosphere-based-global-observations-through-2021-no-18-26-october-2022.

[B162] World Meteorological Organization (2024). “ Greenhouse Gas concentrations hit record high,” in Again. Available online at: https://wmo.int/news/media-centre/greenhouse-gas-concentrations-hit-record-high-again.

[B163] Wu H. Chen S. Ding J. Tian W. Wang T. Shen L. . (2022). New finding of Trichoderma asperellum in decreasing soil N_2_O emission. Chem. Biol. Technol. Agric. 9, 77. doi: 10.1186/s40538-022-00338-8

[B164] Xu Z. Zhou G. (2008). Responses of leaf stomatal density to water status and its relationship with photosynthesis in a grass. J. Exp. Bot. 59, 3317–3325. doi: 10.1093/jxb/ern185 18648104 PMC2529243

[B165] Yamulki S. Holt A. (2017). “ Tree emissions of CH_4_ and N_2_O: Briefing and review of current knowledge,” in The Reseach Agency of the Forestry Commission ( The reseach agency of the forestry commission, London, UK). Available online at: https://cdn.forestresearch.gov.uk/2022/02/tree_emission_of_ch4_and_n2o_sirwan_yamulki_oct2017-online.pdf.

[B166] Yang L. Zhang X. Ju X. (2017). Linkage between N_2_O emission and functional gene abundance in an intensively managed calcareous fluvo-aquic soil. Sci. Rep. 7, 43283. doi: 10.1038/srep43283 28233823 PMC5324132

[B167] Yang Y. Zhang H. Shan Y. Wang J. Qian X. Meng T. . (2019). Response of denitrification in paddy soils with different nitrification rates to soil moisture and glucose addition. Sci. Total Environ. 651, 2097–2104. doi: 10.1016/j.scitotenv.2018.10.066 30321731

[B168] Yao R. J. Li H. Q. Yang J. S. Wang X. P. Xie W. P. Zhang X. (2022). Biochar addition inhibits nitrification by shifting community structure of ammonia-oxidizing microorganisms in salt-affected irrigation-silting soil. Microorganisms 10. doi: 10.3390/microorganisms10020436 35208890 PMC8878283

[B169] Yong Z. J. Lin W. J. Lin C. W. Lin H. J. (2024). Tidal influence on carbon dioxide and methane fluxes from tree stems and soils in mangrove forests. Biogeosciences 21, 5247–5260. doi: 10.5194/bg-21-5247-2024

[B170] Yu K. Chen G. (2009). “ Nitrous oxide emissions from terrestrial plants: observations, mechanisms and implications,” in Nitrous Oxide Emissions Research Progress (New York: Nova Science Publishers Hauppauge), 85–104.

[B171] Yu K. W. Wang Z. P. Chen G. X. (1997). Nitrous oxide and methane transport through rice plants. Biol. Fertil. Soils 24, 341–343. doi: 10.1007/s003740050254 30311153

[B172] Zakir H. Subbarao G. V. Pearse S. J. Gopalakrishnan S. Ito O. Ishikawa T. . (2008). Detection, isolation, and characterization of a root-exuded compound, methyl 3-(4-hydroxyphenyl) propionate, responsible for biological nitrification inhibition by sorghum (Sorghum bicolor). New Phytol. 180, 442–451. doi: 10.1111/j.1469-8137.2008.02576.x 18657214

[B173] Zhang Y. Cai Z. Zhang J. Müller C. (2023). Effect of labile and recalcitrant carbon on heterotrophic nitrification in a subtropical forest soil. Eur. J. Soil Sci. 74, e13389. doi: 10.1111/ejss.13389 40046247

[B174] Zumft W. G. (1997). Cell biology and molecular basis of denitrification. Microbiol. Mol. Biol. Rev. 61, 533–616. doi: 10.1128/mmbr.61.4.533-616.1997 9409151 PMC232623

